# Transcription‐coupled repair: tangled up in convoluted repair

**DOI:** 10.1111/febs.70104

**Published:** 2025-04-24

**Authors:** Diana A. Llerena Schiffmacher, Yun Jin Pai, Alex Pines, Wim Vermeulen

**Affiliations:** ^1^ Department of Molecular Genetics Erasmus MC Cancer Institute, Erasmus University Medical Center Rotterdam The Netherlands; ^2^ Master Scientific Illustrations, Department of Anatomy and Embryology, Faculty of Health, Medicine and Life Sciences Maastricht University The Netherlands; ^3^ Present address: Developmental Biology and Cancer UCL GOS Institute of Child Health London UK

**Keywords:** neurodegeneration, TC‐NER hereditary disorders, transcription stress, transcription‐blocking lesions, transcription‐coupled nucleotide excision repair

## Abstract

Significant progress has been made in understanding the mechanism of transcription‐coupled nucleotide excision repair (TC‐NER); however, numerous aspects remain elusive, including TC‐NER regulation, lesion‐specific and cell type‐specific complex composition, structural insights, and lesion removal dynamics in living cells. This review summarizes and discusses recent advancements in TC‐NER, focusing on newly identified interactors, mechanistic insights from cryo‐electron microscopy (Cryo‐EM) studies and live cell imaging, and the contribution of post‐translational modifications (PTMs), such as ubiquitin, in regulating TC‐NER. Furthermore, we elaborate on the consequences of TC‐NER deficiencies and address the role of accumulated damage and persistent lesion‐stalled RNA polymerase II (Pol II) as major drivers of the disease phenotype of Cockayne syndrome (CS) and its related disorders. In this context, we also discuss the severe effects of transcription‐blocking lesions (TBLs) on neurons, highlighting their susceptibility to damage. Lastly, we explore the potential of investigating three‐dimensional (3D) chromatin structure and phase separation to uncover further insights into this essential DNA repair pathway.

## Graphical abstract

In this review, we discuss recent findings derived from diverse genomic, biochemical and structural, imaging, and functional studies (B–E) that culminated in deep mechanistic insight (A) into the vital cellular process of transcription‐coupled nucleotide excision repair (TC‐NER). With this improved insight, we evaluated the impact of TC‐NER on associated diseases and human health. We further address some remaining questions and challenges (F–G) ahead.

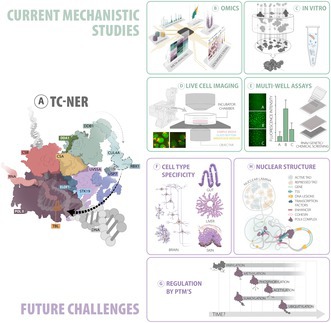

**Abbreviations**
3D: three‐dimensional
6–4PPs: pyrimidine‐pyrimidone (6–4) photoproducts
AI: artificial intelligence
AMeD: aplastic anemia, mental retardation and dwarfism
AP site: apurinic/apyrimidinic site
AP‐MS: affinity purification‐mass spectrometry
ATAC‐seq: assay for transposase‐accessible chromatin using sequencing
BER: base excision repair
CAK: CDK‐activating kinase
ChIP‐seq: chromatin immunoprecipitation‐sequencing
COFS: cerebro‐oculo‐facio‐skeletal
CPDs: cyclobutane pyrimidine dimers
CRL4^CSA^
: CUL4A/B‐RBX1‐DDB1‐DDA1 E3 ubiquitin ligase
Cryo‐EM: cryo‐electron microscopy
CS: Cockayne syndrome
CSA: Cockayne syndrome protein A
CSB: Cockayne syndrome protein B
CSN: COP9 signalosome
DDA1: DET1 and DDB1 associated 1
DDR: DNA damage response
DIvA: DSB inducible via AsiSI
DPC: DNA‐protein crosslink
ds: double‐stranded
DSB: double‐strand break
DSBR: DSB repair
EC: elongation complex
ELOF1: pol II elongation factor 1
FA: Fanconi anemia
FRAP: fluorescence redistribution after photobleaching
GG‐NER: global genome NER
HR: homologous recombination
ICL: interstrand crosslink
ICLR: ICL repair
IDRs: intrinsically disordered regions
IP: immunoprecipitation
iPCs: induced pluripotent stem cells
IR: ionizing radiation
KI: knock‐in
KO: knockout
LP‐BER: long‐patch‐BER
Neddylation: NEDD8 binding
NER: nucleotide excision repair
NPS: non‐photosensitive
PAF1C: PAF1 complex
PARylation: poly(ADP)‐ribosylation
Pol II: RNA polymerase II
PS: phase separation
PTM's: post‐translational modifications
RNAi: RNA interference
ROS: reactive oxygen species
RPS: recovery of protein synthesis
RRS: RNA synthesis recovery
SAR‐seq: synthesis associated with repair sequencing
SP‐BER: short‐patch‐BER
SSB: single‐strand break
SSBR: SSB repair
STK19: serine threonine kinase 19
TADs: topologically associating domains
TBL: transcription‐blocking lesion
TC: transcription‐coupled
TFIIH: general transcription factor II H
TFIIS: transcription factor II S
TIR: TFIIH‐interacting region
TLS: translesion synthesis
TSS: transcription start site
TTD: trichothiodystrophy
UBD: ubiquitin‐binding domain
UDS: unscheduled DNA repair synthesis
USP7: ubiquitin‐specific protease 7
UV: ultraviolet
UVSS: UV‐sensitive syndrome
UVSSA: UV‐stimulated scaffold protein A
WH: winged‐helix
XFE: xeroderma pigmentosum F‐Ercc1
XP: xeroderma pigmentosum
XP‐V: XP variant
XR‐seq: excision repair sequencing

## Introduction

Ensuring accurate gene transcription by RNA polymerase II (Pol II) is essential for maintaining cellular function and survival. Timely gene expression requires tight, multi‐level regulation of the transcription process [1, 2, 3] and an undamaged template strand. However, DNA integrity is challenged by a variety of cell‐intrinsic and environmental DNA‐damaging processes, causing a DNA damage load of approximately 10^5^ lesions a day in each cell [4]. DNA lesions strongly disturb replication processivity and fidelity, resulting in mutagenesis and genome instability. Moreover, DNA lesions are causative of severe transcription stress, including suppressed gene expression, expression of mutant and truncated transcripts, and stalling of the polymerase at a transcription‐blocking lesion (TBL) [5, 6, 7, 8] (Table 2). When stalling occurs, the newly synthesized RNA may hybridize with the single‐stranded DNA, forming deleterious R‐loops in which the DNA is more susceptible to modifications [9, 10]. TBL‐induced R‐loop formation was found to stimulate ATM kinase activation, which is important for the cellular response to DNA damage [11]. If not repaired in time, TBL‐bound Pol II and R‐loops form roadblocks for the incoming transcription machinery and the replication fork, constituting a source of transcription and replication stress, ultimately leading to genome instability [7, 12].

DNA modifications are inevitable; they are introduced by various cell‐intrinsic processes, including spontaneous nucleotide depurination or deamination, nucleotide misincorporation during replication, metabolic (by)products such as reactive oxygen species (ROS) and aldehydes, or exogenous DNA‐damaging agents [8, 13, 14, 15]. Minor, nonbulky, nucleotide modifications such as base oxidation and alkylation induce genomic and transcriptional mutagenesis, but typically do not fully block Pol II transcription [13, 16]. In contrast, abasic sites produced by DNA hydrolysis and structurally altered DNA, such as intra‐ and inter‐strand crosslinks, severely compromise efficient elongation, leading not only to replication‐associated mutations but also to severe changes in the transcriptome. This TBL‐induced transcription stress possibly triggers cellular dysfunction, senescence, and cell death, all of which are hallmarks of aging [14, 17]. Additionally, the DNA structure is also altered by a plethora of different environmental mutagens, such as ultraviolet (UV) and ionizing (IR) radiation, chemicals, and chemotherapeutic drugs that can chemically modify nucleotides (oxidation, alkylation, DNA adducts), intercalate between base pairs, or cause DNA strand breaks [6, 8, 18]. UV light was found to predominantly introduce helix‐distorting lesions, including cyclobutane pyrimidine dimers (CPDs) and pyrimidine‐pyrimidone (6–4) photoproducts (6–4PPs), which bend the DNA at a 9° and 44° angle, respectively, and impair the incorporation of the ribonucleotide, thereby causing transcriptional arrest [6, 19].

In response to these genomic insults, cells initiate a series of protective mechanisms collectively known as DNA damage response (DDR). DDR modulates cellular processes via chromatin modifications, checkpoint activation, transcriptome and proteome alterations, and mobilizes DNA repair factors to the site of damage, resulting in the removal of the stalled polymerase and the repair of the lesion [20]. The importance of genome maintenance is highlighted by a broad range of DNA repair mechanisms that are evolutionarily conserved, including single‐ or double‐strand break (SSB or DSB) repair (SSBR or DSBR), base excision repair (BER), DNA‐protein crosslink (DPC) repair, interstrand crosslink (ICL) repair, and nucleotide excision repair (NER) [21, 22, 23, 24, 25, 26]. While DNA repair pathways such as BER employ lesion‐specific glycosylases [27], global genome (GG‐)NER scans the DNA to identify damage‐induced distortions in the DNA helix, rather than the DNA lesion itself. This results in the recognition of a broad range of DNA lesions throughout the genome by GG‐NER [28, 29, 30]. To secure fast lesion removal in actively transcribed genes, lesion‐stalled Pol II is sensed through specialized proteins. These proteins form part of transcription‐coupled (TC) repair pathways recognizing NER [7, 28], BER [31], DSBR [32, 33], DPC repair [34, 35, 36], and ICL repair substrates [37, 38, 39]. While damage recognition in GG‐NER and TC‐NER is distinct, both pathways process the lesion uniformly by verifying the DNA adduct through general transcription factor II H (TFIIH)—a multifunctional complex comprising seven core subunits including XPB, XPD, and three CDK‐activating kinase (CAK) components [40, 41, 42]—and cutting the damaged strand by endonucleases ERCC1‐XPF and XPG. DNA synthesis and strand ligation restores the DNA to its original state [28].

## Current model

The most studied transcription‐associated repair system is the strand‐specific TC‐NER pathway, engaging in its core complex Cockayne syndrome proteins A and B (CSA and CSB), and UV‐stimulated scaffold protein A (UVSSA) (Table 1) [7, 28, 43]. CSB, a SWI2/SNF2‐like ATPase, is considered the first responder, binding the DNA upstream of the blocked polymerase and causing the DNA to bend approximately 50° [44]. ATP binding initiates the translocation of CSB, which simultaneously causes the forward movement of Pol II. CSB thereby pushes DNA lesion‐paused Pol II over non‐bulky endogenous damage and prevents transcription stalling and premature termination by these minor lesions [44, 45, 46]. However, upon TBL encounter, CSB is unable to translocate the polymerase and instead firmly locks Pol II onto the lesion [44]. Lesion‐stalled and CSB‐locked Pol II prevents access of the DNA repair machinery and thus requires significant remodeling, which will be further discussed in section “From lesion recognition to removal”. This CSB‐Pol II complex stimulates the incorporation of CSA into the TC‐NER complex via the CSA‐interacting motif of CSB [43, 47]. CSA is a WD40 DCAF protein that functions as substrate receptor for the CUL4A/B‐RBX1‐DDB1‐DDA1 E3 ubiquitin ligase (CRL4^CSA^), important for the ubiquitylation of CSB and Pol II during TC‐NER [48, 49, 50, 51, 52]. Ubiquitylation activity requires a conformational change of the cullin backbone, achieved by the binding of ubiquitin‐like protein NEDD8 (neddylation) [48, 49, 53]. Core TC‐NER factor UVSSA is a highly flexible protein containing multiple domains that are important for its interaction with CSA and the recruitment to DNA damage sites [43, 46, 54, 55, 56]. Recent cryo‐electron microscopy (EM) studies revealed more details about the C‐terminal end of the protein and its function during TC‐NER [53] (discussed in section “The architecture of TC‐NER factors interacting with Pol II”). UVSSA also interacts with ubiquitin‐specific protease 7 (USP7), a de‐ubiquitylating enzyme. USP7 binding stabilizes UVSSA and counteracts UV‐induced CSB ubiquitylation, thereby preventing VCP/p96‐mediated proteasomal degradation of CSB [57, 58, 59]. Moreover, UVSSA is essential for the recruitment of TFIIH (discussed in section “From lesion recognition to removal”) and thus secures the continuation of NER [43, 55, 60]. While significant progress has been made recently in understanding the TC‐NER mechanism, numerous aspects remain elusive. Here, we summarize recent developments and remaining challenges in the field.

**Table 1:** (TC‐)NER protein components.

Protein (complex)	Yeast homolog	Function in (TC‐)NER	Reference
RNA polymerase II^a^	RNA polymerase II^b^	TBL recognition	[334]
CSB	Rad26	Sensing of stalled Pol II	[335]
CSA	Rad28	Substrate receptor of CRL4^CSA^	[336]
DDB1	Ddb1	CRL4 component	[49]
DDA1	—	CRL4 component	[52]
CUL4A/B	Cul4	CRL4 component	[49]
RBX1	Hrt1	CRL4 component	[49]
NEDD8	Rub1	CRL4 activity	[48, 78]
COP 9 signalosome (CSN)^c^	CSN^d^, ^e^	CRL4 activity	[48, 49, 78, 79]
CCT/TRiC complex^f^	CCT complex^g^	CSA folding	[74]
ELOF1	Elf1	UVSSA and TFIIH recruitment	[61, 62]
UVSSA	—	Complex stability & USP7 and TFIIH recruitment	[57, 58, 60]
USP7	—	De‐ubiquitylation and protection of CSB	[57, 58]
SPT16	Spt16	UVSSA recruitment	[56]
STK19	—	CRL4^CSA^ activity & TFIIH anchoring	[65, 66, 67, 68]
TFIIH complex^h^	TFIIH complex^i^	Lesion verification	[140, 141, 185]
XPA	Rad14	Lesion verification and assembly of NER incision complex	[141]
RPA^j^	Rpa^k^	Stabilization of non‐damaged DNA strand	[143]
XPF‐ERCC1	Rad1–Rad10	5′ end incision of damaged DNA strand	[142, 143]
XPG	Rad2	3′ end incision of damaged DNA strand	[142, 143]
HLTF	Rad5	Removal of damaged DNA strand	[146]
RFC^l^	Rfc^m^	Loading and unloading PCNA onto DNA	[144, 145]
PCNA	Pcna (Pol30)	Scaffold to recruit DNA polymerases	[144, 145]
DNA polymerases δ, ε, or κ	DNA polymerases δ (Pol3), ε (Pol2), or κ (Mug40)^n^	DNA synthesis of new DNA strand	[144, 145]
DNA ligase I	DNA ligase I	Ligation of new DNA strand	[144, 145]
PAF1 complex^o^	Paf1 complex^p^	Transcription restart after repair	[73]

^a^ Human RNA pol II subunits: RPB1–RPB12.

^b^ Yeast RNA pol II subunits: Rpb1–Rpb12.

^c^ Human CSN: CSN1–6; CSN7A; CSN7B; CSN8.

^d^ Yeast CSN: Csi1; Csn5 (Rri1); Csn9; Csn10 (Rri2); Csn11 (Pci8); Csn12.

^e^ Present in *Saccharomyces cerevisiae* but not found in *Schizosaccharomyces pombe*.

^f^ Human CCT/TRiC complex: TCP1; CCT2–8.

^g^ Yeast CCT/TRiC complex: Cct1–8.

^h^ Human TFIIH complex: XPB; XPD; GTF2H1; GTF2H2; GTF2H3; GTF2H4; GTF2H5; MAT1; CCNH; CDK7.

^i^ Yeast TFIIH complex: Rad52 (Ssl2); Rad3; Tfb1; SSL1; Tfb4; Tfb2; Tfb5; Tfb3; Ccl1; Kin28.

^j^ Human RPA subunits: RPA1–3.

^k^ Yeast RPA subunits: Rfa1–3.

^l^ Human RFC subunits: RFC1–5.

^m^ Yeast Rfc subunits: Rfc1–5.

^n^ Present in *Schizosaccharomyces pombe* but not found in *Saccharomyces cerevisiae*.

^o^ Human PAF1 complex: PAF1; CDC73; LEO1; CTR9; WDR61.

^p^ Yeast Paf1 complex: Paf1; Cdc73; Leo1; Rtf1; Ctr9.

## New TC‐NER factors

**Fig. 1:**
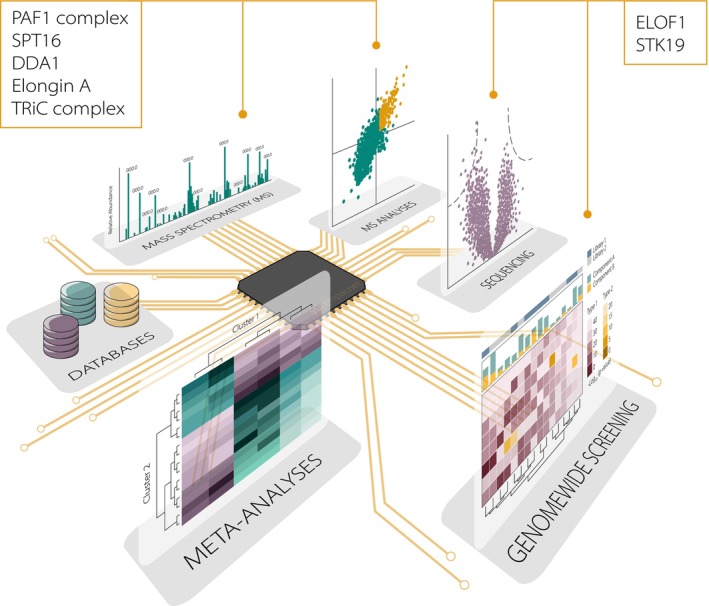
Current Omics approaches to elucidate transcription‐coupled nucleotide excision repair (TC‐NER). Mechanistic studies performed in recent years have focused on utilizing multiple Omics disciplines including proteomics, genomics, and transcriptomics. The fast‐developing mass spectrometry (MS) field has contributed greatly to the discovery of new players (PAF1 complex; SPT16; DDA1; Elongin A; TRiC complex) and of the molecular mechanisms in TC‐NER. Capturing genome‐wide TC‐NER activity has been made possible thanks to advancements in strand‐specific sequencing methods, including eXcision Repair‐sequencing (XR‐seq) [131] and RNA polymerase II (Pol II) chromatin immunoprecipitation (ChIP)‐seq [50]. Furthermore, sequencing of multiple genome‐wide dropout screens performed with a variety of genotoxic substances provided new research targets (ELOF1 and STK19) and shed light on DNA repair‐related pathways. Open access to all the genomic, transcriptomic, and proteomic data stored in different databases is crucial for data integration studies [71] and meta‐analyses, and ultimately a more comprehensive understanding of the underlying mechanisms.

With the discovery of the last UV‐hypersensitivity disease‐causing and TC‐NER‐associated factor, UVSSA, over 10 years ago, multiple research groups have shifted their focus to identifying novel proteins crucial for the TC‐NER reaction. Genome‐wide siRNA and CRISPR‐dropout screens in concert with genotoxic agents and genomic sequencing have uncovered a list of genes that, upon their loss, either induce cellular sensitivity or resistance to DNA‐damaging agents (Fig. 1) [61, 62, 63]. One such factor is Pol II elongation factor 1 (ELOF1), which was found to stimulate TC‐NER through binding at the DNA entry tunnel of the polymerase and facilitating UVSSA and TFIIH recruitment [53, 61, 62, 64]. Additionally, ELOF1 drives TC repair by facilitating the DNA damage‐induced ubiquitylation of lysine K1268 of Pol II's largest subunit RPB1 [50, 61, 62]. The role of K1268 ubiquitylation in TC‐NER will be discussed in more detail in section “Pol II ubiquitylation and beyond: Insights into TC‐NER regulation”. Moreover, ELOF1 has been found to safeguard transcription and genome stability beyond TC‐NER by preventing transcription‐mediated replication stress [62]. A second factor, for which its function in TC‐NER was recently elucidated, is the 29 kDa protein serine threonine kinase 19 (STK19) [65, 66, 67, 68, 69]. In contrast to its name, the protein showed no kinase activity [70], but is an integral component of the TC‐NER protein complex, important for stable UVSSA and TFIIH binding and the timely removal of poly‐ubiquitylated Pol II (discussed in more detail in section “Pol II ubiquitylation and beyond: Insights into TC‐NER regulation”, “The architecture of TC‐NER factors interacting with Pol II” and “From lesion recognition to removal”).

Another powerful method to reveal novel TC‐NER factors is to systematically identify interactors of established TC‐NER factors through affinity purification coupled to mass spectrometry (AP‐MS). Pulldowns of RPB1, CSB, CSA, and UVSSA unveiled multiple (UV‐induced) interactors that modulated DNA repair or transcription restart, including the PAF1 complex (PAF1C), chaperonin TRiC, FACT subunit SPT16, and CRL4^CSA^ interactor DDA1 [43, 52, 56, 71, 72, 73, 74]. While previous data showed PAF1C promoting Pol II elongation in non‐challenged conditions [75, 76, 77], van den Heuvel *et al*. (2021) uncovered that the UV‐induced and CSB‐dependent PAF1C‐Pol II interaction was required for efficient transcription recovery from promoter‐proximal sites following DNA damage‐induced gene silencing. Moreover, pulldowns with GFP‐tagged CSA identified the TRiC chaperonin complex as a CSA interactor, ensuring proper CSA folding and CRL4^CSA^ assembly. Loss of TRiC resulted in reduced nuclear CSA levels and impaired transcription recovery after UV damage, pointing towards TC‐NER defects [74]. On the other hand, studying the role of UVSSA uncovered SPT16, a subunit of H2A/H2B chaperone FACT, as a UVSSA‐interacting protein. While SPT16 recruitment was shown to be UVSSA‐independent, SPT16 was found to facilitate UVSSA recruitment but not CSB. This may indicate a role for SPT16 during TC‐NER complex assembly [56].

AP‐MS using an mClover‐tagged CSA knock‐in (KI) cell line recently discovered a new role for the DET1 and DDB1 associated 1 (DDA1) protein as a CSA‐interacting protein [52]. This protein is a component of the CRL4^CSA^ ubiquitin ligase, modulating its activity and promoting lesion removal by TC‐NER. The loss of DDA1 led to increased UV sensitivity and impaired nascent RNA transcription resumption. Interestingly, MS of tagged CSA in the presence or absence of DDA1 revealed significant alteration in CRL4^CSA^ complex composition under non‐ and UV‐treated conditions. In the absence of DDA1, non‐irradiated CSA exhibited enhanced interactions with TC‐NER components CSB and UVSSA, the CRL complex, and the COP9 signalosome (CSN), which is important for regulating CRL ubiquitin ligase activity through CUL4A/B de‐neddylation [48, 49, 78, 79]. UV irradiation further increased CSA interaction with the TC‐NER complex. Two possible, not mutually exclusive, scenarios were proposed to explain the observations: (a) DDA1 may play a role in maintaining the balance between complex components; and (b) DDA1 might be involved in the dynamic turnover of CRL‐CSN and pre‐assembled CSB‐CSA‐UVSSA. With the plethora of newly identified putative TC‐NER targets (Table 1), and likely more to follow, upcoming publications are expected to shed light on a subset of these targets and elucidate their roles during repair.

## Pol II ubiquitylation and beyond: insights into TC‐NER regulation

The persistent stalling of Pol II at TBLs and the accompanying transcription stress triggers strong DDR signaling, which coordinates key cellular processes via post‐translational modifications (PTM's) [80]. Phosphorylation, poly(ADP)‐ribosylation (PARylation), SUMOylation, methylation, acetylation, and ubiquitylation are examples of PTM's that not only regulate timely DNA repair factor interaction and/or dissociation but also control and silence gene expression directly or through chromatin modifications to protect cells against genomic insults. In addition to PTMs, transcription is also silenced by R‐loop‐mediated ATM signaling at TBLs [11] and GSK3‐mediated Pol II degradation [81], which reduces the promoter‐bound fraction of Pol II genome‐wide. Both topics extend the scope of this review and will not be discussed in detail.

In recent years, several labs have primarily concentrated on Pol II ubiquitylation as a crucial step in TC‐NER. Here, we summarize the key findings of this modification and its function within TC‐NER regulation. Ubiquitylation is involved in a variety of proteolytic and non‐proteolytic cellular functions [82]. The small ubiquitin protein is covalently attached to lysine residues within the target protein, either as mono‐ or poly‐ubiquitin chains through a multi‐step process involving diverse ubiquitin conjugating and ligating activities. While a single ubiquitin changes the protein's interaction profile [83], poly‐ubiquitin chains serve as signaling tags [84, 85, 86] or mark the protein for proteasomal degradation [87]. The outcome depends on which lysine residue in the ubiquitin molecule the chain is formed (e.g., K6; K48; K63) [82]. The inducibility, reversibility, and recognition of ubiquitin by specialized domains make it a versatile dynamic signaling mechanism suitable for highly complex and adaptable processes such as DNA repair.

Numerous MS techniques have been developed that provided valuable insights into the ubiquitylation mechanisms governing the cellular response to UV irradiation [71, 88, 89]. Recent studies have shown that RPB1 is a key target for ubiquitin‐dependent regulation of transcription elongation. Specifically, ubiquitylation of lysine residue K1268 was found to play a crucial role in TC‐NER by stimulating UVSSA and TFIIH recruitment [50, 51]. Although the majority of RPB1 K1268 ubiquitylation has been attributed to the CRL4^CSA^ complex [50], the precise molecular mechanism remains a subject of debate as a part of the TBL‐induced RPB1 ubiquitylation may occur by a CSA‐independent event [51]. Several recently identified TC‐NER factors were found to be linked to CRL^CSA^‐mediated Pol II ubiquitylation, including ELOF1, STK19, and DDA1 [52, 62, 65, 67]. With ELOF1 being important for UVSSA and TFIIH recruitment, it was also shown that ELOF1 was crucial for proper UV‐induced RPB1 ubiquitylation [61, 62]. Similarly, the ubiquitin signaling profile of UV‐treated STK19 knockout (KO) cells showed a clear reduction of RPB1‐K1268 ubiquitylation, along with other RPB1 ubiquitylation sites, which is in line with STK19's stimulating effect on CLR4^CSA^ activity [65]. In contrast, pull‐down experiments for mono‐ and poly‐ubiquitylated proteins in wild‐type and STK19 KO cells indicated that initial UV‐induced Pol II ubiquitylation remained the same but that STK19 KO cells displayed increased Pol II ubiquitylation at later time points [67]. Follow‐up experiments will be needed to pinpoint STK19's function on Pol II ubiquitylation. It is likely that incorporation of ELOF1, STK19, and DDA1 modulates the architecture of the TC‐NER complex to expose the K1268 site and/or allow access of CRL4^CSA^ to Pol II. An interesting reciprocal regulation is noted: as TC‐NER factors are required for efficient K1268 ubiquitylation, so too does ubiquitylation appear important for stable association of some TC‐NER factors [50]. This sequential cooperativity was explained by the mutual dependency of both UVSSA and STK19 for proper TC‐NER complex assembly [65]. Besides complex stability, (poly)‐ubiquitylation of Pol II may also be required for the degradation and thereby removal of lesion‐bound Pol II, as shown by K48‐linked proteolytic ubiquitin chains on RPB1 K1268. Strikingly, also signaling K63‐linked ubiquitin chains were found on RPB1 K1268 [50, 53]. Indeed, flow cytometry experiments showed that Pol II degradation was greatly reduced in STK19‐depleted cells [65]. Treatment of UV‐irradiated and STK19‐depleted cells with transcription inhibitors (thus reducing the number of trailing Pol II) revealed that STK19 was essential for the timely clearance of TBL‐stalled Pol II, thereby facilitating TC‐NER continuation and emphasizing how single TC‐NER components can modulate the repair reaction by changing ubiquitylation [65, 67]. Together, these findings put CRL^CSA^ in the driver's seat for K1268 ubiquitylation. However, recent analysis on the role of the newly identified CRL4^CSA^ component DDA1 indicates that this CSA‐dependent RPB1 modification appears more complicated than initially anticipated [52]. Strikingly, ubiquitin profiling by MS of CSA KO—and to a lesser extent DDA1 KO—showed already CRL^CSA^‐dependent RPB1‐K1268 ubiquitylation in the absence of exogenously induced DNA damage. This observation confirmed CRL4^CSA^'s role in RPB1‐K1268 ubiquitylation and suggests that the trigger for this activity may arise from persistent albeit low levels of TBLs generated by endogenous sources of DNA damage.

Several other E3 ligases have been reported to act on Pol II, including NEDD4 [90] and the CUL5‐Elongin E3 ligases [91, 92], both of which are part of the so‐called “last resort pathway” [93] that clears lesion‐stalled Pol II (discussed in section “From lesion recognition to removal”) when TC‐NER fails. These ligases work in synchrony in a two‐step process for Pol II poly‐ubiquitylation. NEDD4 first mono‐ubiquitylates Pol II, and then CUL5‐Elongin poly‐ubiquitylates it, targeting Pol II for p97/VCP‐dependent extraction and proteasomal degradation [81, 91]. Recently, UBAP2/UBAP2L has been found to play a crucial role in this process by acting as a bridge between mono‐ and poly‐ubiquitylation of Pol II [94]. The involvement of multiple E3 ligases reveals a complex ubiquitin‐mediated mechanism in promoting the resolution of TBLs and restarting transcription through multiple processes that control Pol II fate. The intricate regulation of these processes may depend on the type of DNA damage, cellular context, stage of the TC‐NER reaction, and cell cycle [34, 35, 36].

Besides Pol II, other TC‐NER proteins such as CSB and UVSSA were found to be ubiquitylated in response to DNA damage [95, 96]. Recent evidence suggests that CRL4^CSA^‐directed ubiquitylation is required for CSB remobilizing from the site of UV‐induced DNA damage. CSB was shown to remain bound to UV‐damaged chromatin 4 h post UV and that the release, rather than the recruitment, of CSB is affected in CSA KO cells [72, 97]. As UVSSA is important for the protection of CSB from UV‐induced degradation [57, 58], both ELOF1 and STK19 were shown to alter CSB degradation, albeit to opposite extents [65]. While ELOF1 depletion accelerated CSB degradation, STK19‐depleted cells displayed lower degradation, explicable by the effect of STK19 on CRL4^CSA^ activity. UVSSA also undergoes ubiquitylation at Lysine 414 (K414) in response to DNA damage, which is dependent on the ubiquitylation of Pol II [50, 96]. While the precise mechanisms by which these modifications regulate each other remain unclear, they both seem to facilitate the transfer of TFIIH from UVSSA to lesion‐stalled Pol II. Besides CSB ubiquitylation, CSB possesses a C‐terminal ubiquitin‐binding domain (UBD), which has been implicated in its dynamic association with damaged chromatin [98].

**Fig. 2:**
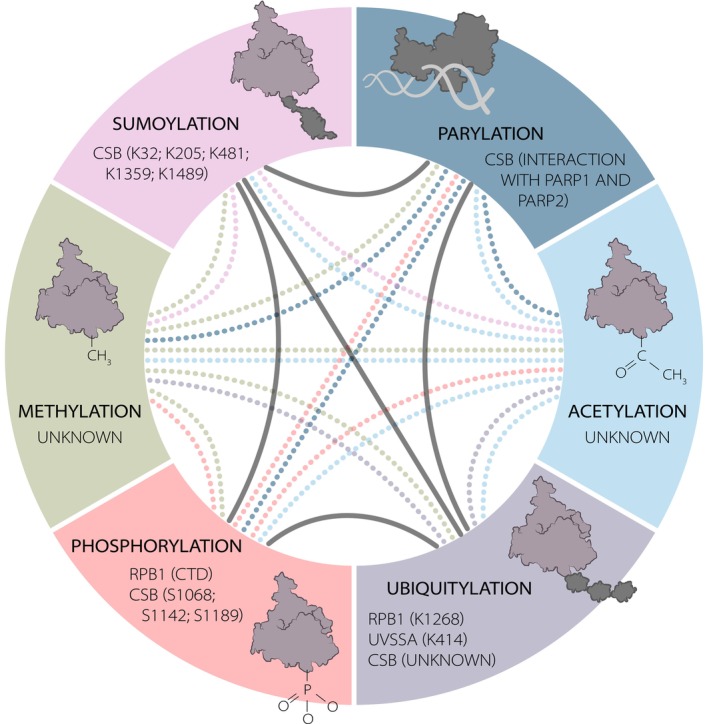
Post‐translational modifications (PTMs) during transcription‐coupled (TC) repair. PTMs, including PARylation [113, 114], ubiquitylation [50, 51, 96], phosphorylation [2, 337] and SUMOylation [100] play an important role during TC repair. Due to their highly dynamic nature, it is currently unknown at what time points or during which stages of repair the PTMs occur. While acetylation and methylation are thus far not directly linked to the TC‐NER machinery, they may play a role in downstream events, including transcription resumption [73, 237, 338, 339]. Previous reports indicated interdependencies of PTMs during DNA repair (indicated by black lines) [99, 100, 101, 102, 340, 341, 342]. Whether this takes place for other PTMs is currently unknown (dotted lines).

Recent discoveries revealed that ubiquitin modifications are highly interconnected with other types of PTMs [99, 100, 101, 102], although their function in TC‐NER is less well described than in GG‐NER [103]. These include phosphorylation [104], acetylation [105, 106, 107, 108, 109], methylation [110, 111], SUMOylation [100], and PARylation [112, 113, 114, 115] of repair factors and chromatin components. Together, these PTMs may provide additional regulatory nodes to maintain cellular homeostasis amidst TBLs. Understanding the relevance and precise roles of each of these dynamic PTMs will be challenging, as many occur transiently, in between mechanistic transitions, and may be highly interdependent (Fig. 2).

## The architecture of TC‐NER factors interacting with Pol II

**Fig. 3:**
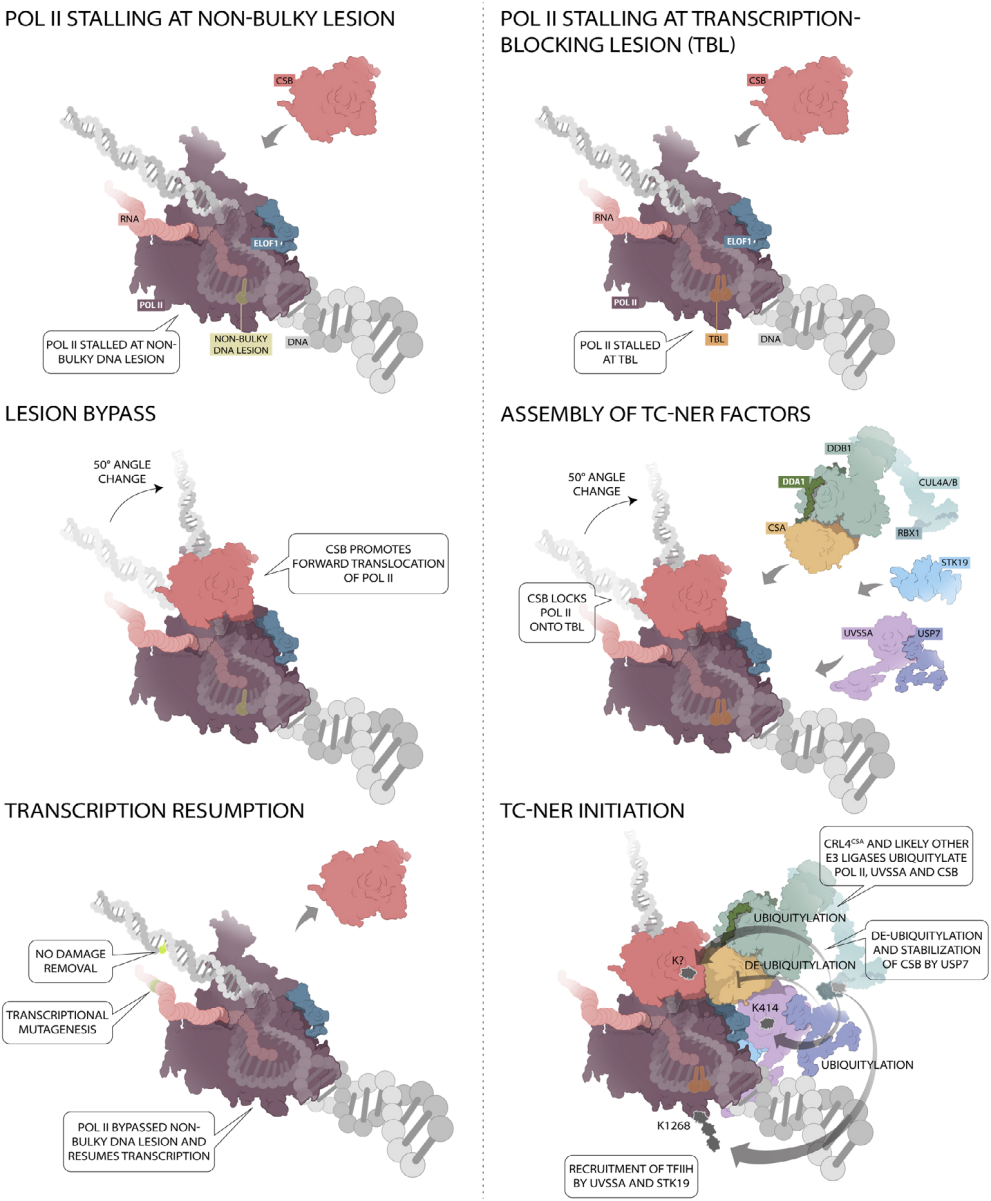
Processing of stalled RNA polymerase II (Pol II) at a non‐bulky or transcription‐blocking lesion (TBL). (Left, from top to bottom) Pol II (plum) traveling together with ELOF1 (dark blue) is stalled at a non‐bulky lesion (green). Lesion stalling is sensed by CSB (red) which binds and subsequently bends the upstream DNA. CSB ATPase activity promotes the forward movement of Pol II, resulting in lesion bypass and transcription resumption. As a consequence, the RNA transcript contains a mutated nucleobase, and the non‐bulky lesion remains in the template DNA. (Right, from top to bottom) Pol II, together with ELOF1, is stalled at a TBL, which is recognized by CSB. Idem, CSB changes the angle of the DNA and initiates Pol II translocation, which in this case locks Pol II onto the lesion rather than moving it past it. TC‐NER complex assembly follows, including CRL4 (CUL4A/B; DDB1; RBX1; DDA1) (green) with receptor adaptor CSA (yellow), UVSSA (light purple), USP7 (dark purple) and STK19 (light blue). TC‐NER initiation includes the regulation of the complex by CRL4‐mediated ubiquitylation of Pol II (K1268) [50, 51], UVSSA (K414) [96], and CSB (lysine residues unknown). CSB ubiquitylation is counterbalanced by USP7. The downstream factor TFIIH is recruited by UVSSA (direct interactions with p62 (*GTF2H1*) [55]) and STK19 (direct interaction with XPD (*ERCC2*) [65, 66, 67, 68]).

Technical breakthroughs have not only advanced genomic and proteomic studies but have also profoundly impacted structural biology. To elucidate TC‐NER complex conformation, recent cryo‐EM studies aided by artificial intelligence (AI) structure prediction algorithms such as AlphaFold [116] have provided high‐resolution images and models of Pol II with TC‐NER factors [65, 66, 67, 68]. This endeavor started out with resolving the structure of yeast Pol II with Rad26 (i.e., the yeast ortholog of CSB) [44], showing that Rad26 binds and bends the DNA upstream of Pol II. Based on additional experimental evidence, a model was forwarded that Rad26 acts by promoting forward translocation of Pol II over natural pause sites and elongation‐interfering non‐bulky DNA lesions (Fig. 3). For bulky lesions that cannot be bypassed (Table 2), the strongly locked Rad26 complex triggered the TC‐NER reaction. More recent studies showed that, indeed, dynamic nucleotide binding at Rad26's ATPase domain provided structural motions in Rad26 to change its interface with Pol II and DNA and thus allow its translocation [117]. Cryo‐EM studies on mammalian reconstituted Pol II‐CSB‐CSA‐DDB1‐UVSSA complexes [46], followed by inclusion of ELOF1 [53], confirmed that CSB binds upstream and alters the direction of upstream DNA by approximately 50° as seen in yeast. Modeling of the complex not only elucidated the spatial arrangement of the proteins but also provided new mechanistic insights. The authors identified a helix–loop–helix element in the second ATP lobe of CSB that inserted itself into the upstream DNA fork, resulting in the sequential interaction between the aromatic side chain of phenylalanine residue (F796) with two neighboring nucleobases of the non‐template DNA strand. This interaction facilitated the pulling of upstream DNA and the forward movement of CSB in an *in vitro* transcription assay. Furthermore, high‐resolution images of the interfaces between the proteins provided structural information to correlate known disease‐causing mutations in TC‐NER to their phenotypes (discussed in section “Clinical consequences of TBL repair deficiencies”). For instance, missense mutations in blades 4 and 5 of CSA's WD40 propeller were predicted to interfere with protein structure integrity and substrate binding [49]. Indeed, most of these missense mutations are located in the CSB‐CSA interface [46, 118], explaining the severe TC‐NER defect. On the contrary, the W361C missense mutation in CSA faces towards UVSSA, resulting in aberrant recruitment and binding of UVSSA, thereby explaining the less detrimental UVSS‐like phenotype (see section “Clinical consequences of TBL repair deficiencies”) observed in the patient carrying this mutation [119].

**Table 2:** Classification of DNA lesions.

DNA lesion	TBL
CPDs	Yes
6‐4PPs	Yes
DPC	Yes
ICL	Yes
AP site	Yes
SSB and DSB	Yes
Oxidated nucleobase	No
Alkylated nucleobase	No
Illudin‐S	Yes
Trabectedin	Yes

Comparing the structure of the canonical elongation complex (EC*)—Pol II‐DSIF‐RTF1‐SPT6‐PAF complex [46]—to the structure containing the TC‐NER core complex (EC^TCR^) revealed a clear conformation shift upon TBL encounter (Fig. 3). Structural analysis, coupled with biochemical assays, demonstrated that CSB and the elongation factor DSIF compete for the same Pol II binding site, suggesting that CSB and DSIF are mutually exclusive. Simultaneous to CSB binding, another elongation factor RTF1 likely dissociates. SPT6 and the PAF complex then change conformation to allow formation of the TC‐NER competent EC^TCR^ complex. Incorporating CRL4^CSA^ into this EC^TCR^ provides the structural basis for two dynamic states: one showing that UVSSA positions RBX1—and its associated E2—towards K1268 of RPB1, and another in which RBX1‐E2 is repositioned to the C terminus of CSB. Although this modeling provided a possible explanation for the notion that this single RBX1‐E2 complex connected by CRL4^CSA^ can reach distant targets, it does not explain how the required substantial rearrangements are achieved to prevent clashes with Pol II. The mechanism triggering the ubiquitylation of this single residue in RPB1 was recently established [53]. Here, ELOF1 was found to play a central role in positioning the CRL4^CSA^ ubiquitin ligase through its interaction with CSA, and in rearranging the TC‐NER complex, which stabilized the previously unstructured C‐terminal part of UVSSA [53]. Collaboratively, ELOF1 and UVSSA facilitate stable binding of CRL4^CSA^ onto stalled Pol II, resulting in efficient K1268 ubiquitylation. Importantly, this role of ELOF1 in stabilizing the TC‐NER complex on Pol II further supports reciprocal cooperativity of the TC‐NER constituents. This refines the previously proposed strict sequential order of assembly in which UVSSA docking is fully dependent on the presence of CSA [43]. Notably, the reconfiguration of the ubiquitin ligase through neddylation of CUL4 played a major role in aligning the donor ubiquitin site closer to the K1268 loop. Interestingly, this new EC^TCR^ structure showed that the C‐terminal part of UVSSA folded back towards the N‐terminal VHS domain, thereby interacting with the downstream DNA, firmly anchoring it in place and blocking the DNA entry tunnel of Pol II. Analogous to DSIF and CSB, UVSSA seems to replace transcription factor II S (TFIIS) located at the DNA entry tunnel during canonical transcription elongation. TFIIS is crucial for 3′ nascent RNA cleavage upon Pol II pausing and subsequent reactivation of the polymerase at natural pause sites [120, 121]. This points towards a new function of UVSSA during the repair process involving the stabilization and reactivation of backtracked Pol II, and thereby rendering the lesion accessible to downstream NER factors.

The most recent accomplishment in resolving the TC‐NER complex structure is the revelation of STK19's three winged‐helix (WH) domain that allows it to interact with multiple proteins, including the clamp element of RPB1, DDB1, CSA, and UVSSA, and with downstream DNA [65, 66, 67, 68]. These interactions contribute to TC‐NER complex stability and repair progression, as highlighted by compromised UVSSA and TFIIH binding in STK19 KO cells [65, 67]. It was further shown that the interaction between STK19 and CSA‐DDB1 is crucial for TC‐NER repair [66]. One of the main mechanistic findings for STK19 is the proposed interaction between STK19 and TFIIH component XPD (discussed in more detail in section “From lesion recognition to removal”). Interestingly, superimposing the TC‐NER structure with the previously resolved TFIIH‐XPA‐DNA complex [122] revealed a structural clash between UVSSA's VHS domain and XPD, suggesting that UVSSA undergoes a conformational change within the complex structure upon arrival of TFIIH [65, 66, 67, 68]. Intriguingly, STK19 was found to change the conformation of the TC‐NER complex, revealing a novel contact point between the flexible C terminus of CSA (W389) and the VHS domain of UVSSA [66]. While mutating this highly conserved tryptophan and the adjacent serine resulted in a loss of mono‐ubiquitylated UVSSA *in vitro*, mutating the previously identified connection between the β‐propeller of CSA and UVSSA showed no effect on UVSSA ubiquitylation. This result is unexpected as the β‐propeller structure provides the substrate binding surface for WD40 proteins [49, 123], suggesting that this new C‐terminal interaction could possibly keep UVSSA within range of efficient ubiquitylation by the CRL4 complex. It might also provide structural flexibility and space for the recruitment of downstream NER factors.

It is striking to note that with every new TC‐NER factor identified and their respective incorporation in architectural models, mechanistic insight is strongly advanced. Additionally, with improved cell‐free TC‐NER assays, functional interpretation of cell biological, genetic, proteomic, and structural findings is strongly increased. This highlights the synergistic strength of integrating molecular biological sub‐disciplines. It is therefore intriguing to speculate on how the *in vitro* constituted TC‐NER protein structure would change upon incorporation of additional components, whether the subsequent factors are additive or in competition with the existing TC‐NER proteins, and at which stage of the repair process they are involved. Such components could be USP7, proteins identified as TC‐NER interactors by MS or inclusion of a TBL‐containing DNA strand, as all Pol II complexes were examined on non‐damaged DNA templates thus far.

## Tools to study TC‐NER

The rapid evolving fields of large‐scale CRISPR‐mediated genetic dropout screens, sophisticated and highly sensitive MS analyses, single particle cryo‐EM and associated AI‐driven molecular modeling of intricate protein complexes have significantly advanced the TC‐NER field (see section “The architecture of TC‐NER factors interacting with Pol II”). Within the last years, multiple new TC‐NER regulatory factors have been identified and a wealth of insightful structures of TC‐NER complexes with increasing complexity appears almost every month. To functionally validate and test the significance of newly identified TC‐NER factors and regulators, only a limited set of elaborate TC‐NER assays are currently available. The most commonly applied assays are mainly cellular biological end‐point tests, such as DNA damage survival assays and indirect monitoring of TC‐NER performance by RNA synthesis recovery (RRS) after DNA damage‐induced transcription inhibition [124, 125]. Current efforts have established a new TC‐NER assay which monitors the recovery of protein synthesis (RPS) after induction of damage. While both RRS and RPS assays capture transcriptional shutdown following damage—either directly or indirectly—RPS stands out for its versatility. This assay can be conducted in various cell types and even in differentiated tissues of living organisms, allowing for real‐time monitoring of TC‐NER activity [126]. To directly measure actual repair capacity a single‐cell imaging‐based TC unscheduled DNA repair synthesis (TC‐UDS) assay was developed, in which the relative low level of TC‐NER‐derived UDS was amplified [127]. This assay, although it requires the depletion of GG‐NER (e.g., XPC KO) whose much higher UDS level would otherwise mask TC‐NER UDS, is highly useful to determine whether new factors are required for TC‐NER or post‐repair transcription resumption [56]. The first, and signature, TC repair assay involved visualizing UV‐lesion removal from either the transcribed or non‐transcribed strand of linearized genomic DNA with strand‐specific probes by Southern blotting [128, 129]. By first incubating UV‐irradiated cells with a pyrimidine dimer‐specific endonuclease [130], this assay indirectly reveals whether repair had taken place and thus, while labor‐intensive, provides reasonable estimates for DNA repair efficiency. The methods to detect repair significantly improved by the development of genomic sequencing techniques, such as eXcision Repair‐sequencing (XR‐seq) [131], or the Pol II chromatin immunoprecipitation‐sequencing (ChIP‐seq) procedure [50, 61]. Both monitor DNA repair at the gene level while taking strand‐specificity into account. For a better mechanistic understanding significant efforts have been invested to set up *in vitro* TC‐NER assays. The Egly lab [132] successfully established a TC‐NER assay; however, this system only managed very low levels of actual repair of the *in vitro* damaged template DNA. Moreover, in this system, the loading of the NER incision machinery appeared CSB‐independent, contrasting to the fully CSB‐dependent TC‐NER complex assembly in cells [43]. This discrepancy is likely caused by the *in vitro* system lacking vital TC‐NER factors not known at the moment. The order of TC‐NER factor recruitment in cells was established by immunoprecipitation (IP) of lesion‐stalled Pol II, suggesting a strictly sequential hierarchical assembly model. As described above, incorporating new factors into sophisticated TC‐NER complex structures refined the model and produced an intricate reciprocal cooperative assembly model in which late‐appearing factors (e.g., UVSSA) stabilize the incorporation of earlier proteins (e.g., CSA). This apparent discrepancy between the models could be attributed to the limitations of the performed chromatin IP assay which cannot clearly distinguish between recruitment or stable binding. This reciprocal complex stabilization is in line with previous live cell imaging studies (explained in section “Live cell TC‐NER monitoring”), showing that UVSSA can be recruited to stalled Pol II independent of CSA [56]. The most recent breakthrough is by the development of a cell‐free TC‐NER assay using nuclear extracts from frog eggs, complemented with transcription stimulating factors and based on monitoring the removal of transcription‐blocking lesions from a plasmid containing a single Cis‐Pt lesion in the transcribed strand [66]. This novel *in vitro* TC‐NER assay may prove valuable in measuring the impact of newly identified TC‐NER factors such as STK19, and to test the effect of specific mutations that may disrupt TC‐NER factor interactions. The main disadvantage of this heterologous system is that these egg extracts have only limited transcription capacity and by inference inefficient TC‐NER and are assayed without the structural nuclear context.

## Live cell TC‐NER monitoring

To monitor NER within the context of intact living cells, live‐cell imaging assays using GFP‐tagged GG‐NER factors were developed approximately 25 years ago. These assays measured either the fluorescence redistribution after photobleaching (FRAP) or the real‐time accumulation of NER proteins binding at locally inflicted DNA damage [133, 134]. These procedures enabled direct measurement of NER and GG‐NER specific factor assembly, protein mobility, and binding to DNA damage‐containing chromatin (i.e., damage‐dependent immobilization in FRAP), allowing a sneak preview of live cell NER kinetics [135]. Unfortunately, this method appeared not sufficiently sensitive to efficiently monitor TC‐NER from ectopically expressed factors, although statistically significant binding kinetics of CSB could be retrieved from large data sets [136]. The advent of CRISPR‐Cas9 enables the generation of fluorescently tagged KI cell lines expressing GFP‐fusion proteins from their endogenous promoter. FRAP experiments with endogenously tagged cell lines offer a highly sensitive method to investigate the impact of TBLs on transcription and TC‐NER, highlighted by the clearly recognizable immobilization of the protein even with low doses of DNA‐damaging agents [52, 72, 81, 97, 137].

GFP‐RPB1 FRAP experiments in combination with computational modeling distinguished four different kinetic pools of Pol II in unchallenged cells by using a set of transcription inhibitors that interfere with different stages of the transcription cycle [137]: a small (7%) free diffusing fraction representing non‐engaged Pol II; a 10% fraction which was transiently chromatin‐bound for 2.4 s, representing dynamic promoter interactions; a 23% fraction with surprising dynamic interactions (on average 42 s) at promoter‐proximal paused sites; and about 60% of a longer immobilized (average of 23 min) chromatin‐bound fraction, representing processive elongation. Using nascent mRNA‐sequencing in the same cell line, the number of actively transcribed genes and average gene length were calculated from which the elongation speed could be deduced. This speed strikingly matched earlier calculations [138] and is in line with the FRAP‐determined chromatin dwell times. These parameters not only show that the GFP‐tagged Pol II is fully functional but also illustrate the validity of this live cell model system to retrieve quantitative information on transcription progression [137]. Treatment of GFP‐RPB1 cells with different doses of UV revealed a dose‐dependent reduction in RPB1 mobility (i.e., increased immobilization) shortly after irradiation [81]. Interestingly, within the first hour, RPB1 fluorescence intensity remained unchanged, indicating that the majority of lesion‐bound Pol II was not targeted for degradation or released from the chromatin but rather served as an anchor for TC‐NER complex assembly. Within a few hours post‐UV (dose‐dependent), mobility parameters returned to those of untreated cells. Importantly, TC‐NER‐deficient cells showed a lack of Pol II remobilization, indicating that the return of Pol II mobility corresponds to TBL resolution and that FRAP‐based assays truly monitor TC‐NER progression. Strikingly, at time points beyond 1 h after irradiation, an apparent stronger immobilization was observed. However, at these time points, a clear Pol II degradation was also detected, which convoluted the FRAP data interpretation. A possible explanation for these observations is the degradation of promoter‐proximal bound Pol II, triggered by a thus far uncharacterized lesion‐stalled Pol II signaling event [81]. Together, these data demonstrate how unconventional live cell imaging procedures may provide direct TC‐NER measurements and also uncover new mechanistic insights that would be difficult to capture using *in vitro* reconstituted assays.

Due to the small percentage of elongating Pol II being stalled at a TBL, the immobile fraction of Pol II after UV irradiation remains relatively small [81]. By contrast, live cell imaging of fluorescently tagged CSB demonstrated strong transcription‐dependent immobilization, even at low doses of UV, that remobilized within 4–16 h post‐irradiation [72, 97]. This indicates that FRAP analysis of TC‐NER factors is not only a fast and highly sensitive method to study chromatin immobilization, but also captures the complete repair mechanism in living cells over time. FRAP provides a platform to measure the effects of various TBL‐causing agents [34, 39, 97], and to determine the impact of (TC‐)NER components on each other. It revealed that CRL4^CSA^ ubiquitin ligase activity and downstream NER factor XPA are essential for CSB release [72]. Interestingly, while 40 to 60% of tagged CSB and CSA immobilized after high UV doses [52, 72, 97], tagged UVSSA responded differently, with only 15–20% of the protein immobilized to lesion‐stalled Pol II [97]. This discrepancy could be attributed to differences in protein levels, shown by an approximately 2‐fold higher fluorescence intensity of UVSSA‐mScarletI compared to CSB‐mScarletI, suggesting that with maximally immobilized CSB, only half of the UVSSA protein pool interacts with CSB while the rest freely diffuses through the nucleus.

**Fig. 4:**
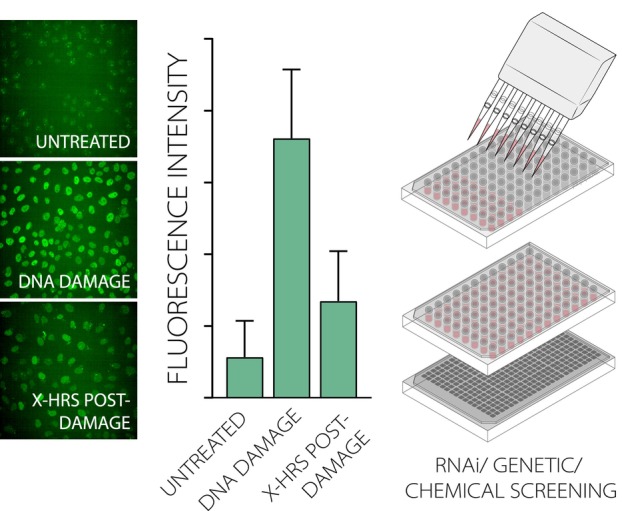
Multi‐well assays measuring DNA repair in bulk promote the identification of new DNA repair factors. Physiological properties such as chromatin binding upon UV‐C damage and the timely release hereof are used to quantitatively screen whether a transcription‐coupled nucleotide excision repair (TC‐NER) protein remains functional when treated with a set of different RNA interference (RNAi) or (chemical) chemotherapeutic drugs targeting candidate TC‐NER modulators. Furthermore, multi‐well assays may allow the identification of proteins that are involved in disease‐associated features, for example, by detecting promotion or alleviation of DDR foci formation (e.g., RAD51 foci, implicated in homologous recombination (HR)). (Left) Fluorescently labeled CSB knock‐in cells [72] revealed a strong nuclear protein retention upon DNA damage, which reduced over time.

Recently, fluorescent CSB KI cells were employed to design a robust single‐cell assay that measured the retention of CSB to the nucleus based on recent FRAP analyses (Fig. 4) [72]. The strong immobilization of fluorescent CSB protein after low doses of UV in live cell imaging suggested that CSB was tightly chromatin‐bound during TC‐NER, which is displayed by a high nuclear fluorescence after pre‐extraction and fixation of the cells. Similarly, previous FRAP results on the mechanistic consequences of CSA or UVSSA loss were reflected by this assay, showing a defect in CSB release upon CSA KO and UV‐dependent CSB degradation in UVSSA KO cells. The assay was developed in a 96‐well format, providing the opportunity to analyze the effect of multiple potential TC‐NER‐regulating proteins, either by knockdown or KO, on CSB function in TC‐NER after UV and possibly after other TBL‐inducing agents. Importantly, assays which can be performed in a multi‐well platform are key for streamlining determination of the functional significance of a large set of potential TC‐NER targets obtained from CRISPR‐dropout screens or MS experiments beyond end‐point (i.e., survival) scoring. They may also facilitate the selection of promising candidates for further investigation in future studies.

## From lesion recognition to removal

As DNA damage sensing is fundamentally different in GG‐NER and TC‐NER [7, 28], it triggers the question of how TC‐NER proceeds to the lesion verification step, in contrast to GG‐NER where this process is rather well‐defined [30, 139]. Among the proteins that are essential for this transition is UVSSA. Besides its function in protecting CSB [57, 58] and thus stabilizing the complete protein complex [72], the protein plays a pivotal role in recruiting TFIIH to lesion‐stalled Pol II. This recruitment is achieved through a direct interaction between the TFIIH‐interacting region (TIR) in UVSSA and the GTF2H1 (p62) component of TFIIH [43, 55]. Intriguingly, the latest identified TC‐NER factor, STK19, provided further insight into this process. STK19's location in front of the stalled polymerase makes it ideal for engaging with TFIIH. Indeed, *in silico* prediction pointed towards a direct interaction between STK19's third WH domain and the helicase XPD [65, 66, 67, 68], which was confirmed by STK19 IP [65]. Additionally, STK19 was found to stimulate TFIIH helicase activity and was shown to modulate TC‐NER complex dissociation [67]. Mutating the three expected STK19‐XPD interfaces individually, and in combination, reduced *in vitro* TC‐NER activity to different extents [66]. Together, this indicates that STK19, in conjunction with the interaction between UVSSA and TFIIH's p62 subunit are crucial for anchoring TFIIH and promoting repair [65, 66, 67, 68].

Functionally, TFIIH comprises several key units including XPB, which serves as a double‐stranded (ds) DNA translocase, and XPD, a 5′–3′ helicase, as well as GTF2H1–5 and the associated trimeric CAK complex (MAT1, CDK7, and CCNH). Together, XPB and XPD are responsible for DNA unwinding during NER and transcription initiation [42]. During lesion verification, XPD plays a central role as it translocates along the DNA in the 5′–3′ direction until encountering the damage, thus confirming its presence [140, 141]. Following verification, TFIIH, assisted by XPA and stabilized through RPA, facilitates the recruitment of structure‐specific endonucleases ERCC1‐XPF and XPG. Interestingly, while ERCC1‐XPF initiates incision at the 5′ site of the TBL, recruitment of XPG 3′ to the lesion is a prerequisite for ERCC1‐XPF engagement [142, 143]. Following 5′ incision but prior to 3′ incision by XPG, PCNA was found to accumulate at sites of UV damage in concert with unscheduled DNA synthesis, thereby protecting the single‐stranded DNA that is created during NER [142]. While the steps involving gap filling and ligation of the newly synthesized DNA strand are less elucidated, the general consensus involves a coordinated action of RFC, PCNA, DNA Polymerases δ, ε, or κ, and DNA ligases [144, 145]. Recent investigations have shed light on these final steps of NER, revealing the additional involvement of RAD5‐related translocase HLTF in actively removing the strand containing the damage and supporting dissociation of the endonucleases [146].

Importantly, while the trimeric CAK sub‐complex of TFIIH is required for transcription initiation through Ser5 phosphorylation of the CTD domain of Pol II [147, 148, 149], the kinase complex was found to inhibit GG‐NER by downregulating XPD helicase activity [122, 150], thus confirming that the dissociation of the kinase complex is required for efficient repair [151]. Additionally, comparing structures of the TFIIH complex during transcription and NER revealed that the interaction domains of the CAK subunit MAT1 and core NER factor XPA shared similar positions within TFIIH, emphasizing that these two proteins need to be exchanged upon TFIIH switching from transcription to DNA repair factor [122]. Strikingly, these findings are in contrast with results obtained with stalled Pol II during TC‐NER. Here, chromatin IP of stalled Pol II revealed a UV‐induced interaction with the CAK subunit CDK7 alongside CSB and CSA, but not with XPA [43, 50]. Moreover, despite the mutual exclusivity of the CAK complex and XPA for interactions with core‐TFIIH, XPA is essential for TC‐NER [28, 141, 152], as its interaction with ERCC1 is required to incorporate the XPF‐ERCC1 endonuclease for the subsequent incision [153]. This was confirmed by showing that XPA KO cells were highly sensitive to Illudin‐S [152] (Table 2), a drug exclusively repaired by TC‐NER [154], and were resistant to the anticancer drug Trabectedin, both of which are specific features of TC‐NER deficiency [155, 156]. Moreover, FRAP experiments showed that in XPA KO cells, CSB remains immobilized several hours after UV irradiation, suggesting a defect in TC‐NER progression [72]. Together, these findings are consistent with a role of the CAK sub‐complex at an early stage, during or simultaneous with the recruitment of TFIIH by UVSSA and the anchoring by STK19, but prior to XPA engagement. Potential roles may include the phosphorylation of other NER factors or the degradation of Pol II, as suggested by reduced Pol II degradation upon CDK7 kinase inhibition and the UV‐induced interaction between Ser5‐phosphorylated Pol II and the ubiquitin ligase receptor Elongin A [157, 158]. However, the exact mechanisms by which TC‐NER utilizes the CAK complex and its timely release to allow XPA incorporation remain unresolved.

The mechanism underlying the processing of lesion‐stalled Pol II remains a significant unanswered question, as the polymerase must somehow be displaced to allow access to the downstream NER machinery, since lesion‐stalled Pol II covers approximately 35 bases around the lesion [6, 159, 160]. One prevailing model suggests that TBLs are exposed through Pol II backtracking by the 5′–3′ translocation of TFIIH [7, 160]. This mode of action would require either CSB release or CSB proteolysis to neutralize the opposite CSB‐mediated pushing force on Pol II. CSB degradation would necessitate the dissociation of the CSB‐protective de‐ubiquitylase USP7 from the TC‐NER complex. Although there is no direct experimental evidence for TFIIH‐mediated CSB release, this hypothesis is supported by live cell imaging data showing persistent chromatin‐bound CSB in XPA KO and TFIIH inhibitor‐treated conditions 4 h post‐UV irradiation [72]. An alternative route to expose the TBLs for repair involves ubiquitin‐mediated Pol II degradation [161]. Supporting evidence for this hypothesis comes from the importance of RPB1‐K1268 ubiquitylation for DNA repair [50, 51], and repair‐independent Pol II degradation in UVSSA KO cells [162, 163, 164]. The latter was shown by a robust VCP‐ and proteasome‐dependent degradation of Pol II after UV‐ or Illudin‐S‐inflicted damage in repair‐deficient UVSSA KO or XPA KO cells. Importantly, this damage‐induced Pol II degradation does not occur in CSA‐ or CSB‐deficient cells, which most likely contributes to the severity of CS, discussed in more detail in section “DNA damage‐induced transcription stress is the main driver of CS”. Additionally, lesion‐stalled Pol II can also trigger the “last resort pathway” [93], in which ubiquitylation and subsequent degradation is controlled by other E3 ligases (as discussed in section “Pol II ubiquitylation and beyond: Insights into TC‐NER regulation”). However, this active degradation‐driven Pol II removal constitutes only a minor fraction of Pol II at TBLs, with most of them being removed by active TC‐NER and may become relevant if TC‐NER is hampered. Alternative theories do not include the removal of TBL‐bound Pol II prior to strand incision by ERCC1‐XPF and XPG, and instead propose that CSB translocation positions Pol II to allow endonuclease activity [165] after which NER‐dependent dual incision removes Pol II together with the excised TBL‐containing oligo. This scenario requires CSB to remodel stalled Pol II in such a way that it allows damage verification by TFIIH, which is essential for endonuclease position. However, structural analysis [44] revealed that CSB locks Pol II more tightly to DNA, making this hypothesis less likely. Additionally, other models including TFIIH enclosing a TBL‐stalled Pol II have been proposed [166].

## Clinical consequences of TBL repair deficiencies

In 1976, Andrews's pioneering work identified NER as the first DNA repair pathway linked to severe neurological abnormalities and a phenotype reminiscent of premature aging [167]. Since then, our understanding of the NER pathway's critical importance has grown substantially. Autosomal recessive mutations in NER‐related genes have been shown to manifest in a diverse array of clinical conditions, including cutaneous UV sensitivity, an increased susceptibility to cancer, progressive neurological decline, and early aging. Phenotypic diversity associated with inherited NER mutations is illustrated by the different syndromes in which patients can be categorized, ranging from xeroderma pigmentosum (XP) to Cockayne syndrome (CS), UV‐sensitive syndrome (UVSS), cerebro‐oculo‐facio‐skeletal (COFS) syndrome, and a subgroup of photosensitive trichothiodystrophy patients [168, 169]. This intricate clinical diversity can be attributed to the complexity of the NER system, which utilizes both GG‐NER and TC‐NER to identify and repair a wide range of DNA lesions and potential other roles of specific factors beyond NER [170].

An illustration of the clinical ramifications of NER deficiency is found in XP, a rare autosomal recessive genetic disorder arising from defective GG‐NER or impaired translesion synthesis (TLS) [171]. Mutations in genes spanning *XPA* to *XPG* result in hypersensitivity to sunlight, skin pigmentation abnormalities, a significantly elevated risk of melanoma and non‐melanoma skin cancers, and neurological impairments, particularly in patients with the more severe form of XP, known as De Sanctis‐Cacchione syndrome [172, 173]. XP also presents a variant form (XP variant, XP‐V), wherein patients maintain NER proficiency but harbor defects in the Y‐family TLS DNA polymerase POLH, resulting in the inability to accurately bypass UV‐induced CPDs and an increased skin cancer risk similar to XP patients [174]. While the cutaneous aspects of XP can be attributed to UV exposure, part of the underlying causes of neurodegeneration in XP was thought to derive from endogenously produced 8,5′‐cyclo‐2′‐deoxyadenosine that were found to accumulate in the brain of *Xpa* ^*−*/−^‐deficient mice over time [175, 176]. Moreover, the clinical severity of XP varies based on the mutation type and the affected gene [177], raising fundamental questions regarding causal mechanisms [178]. For example, missense mutations in XPG result in milder neurological consequences, whereas patients with homozygous mutations causing truncated XPG proteins or complete XPG loss experience more severe neurological symptoms [179], potentially due to XPG's involvement in other NER‐unrelated processes [179, 180, 181, 182, 183, 184]. Similarly, NER factors like helicases XPB and XPD (subunits of repair/transcription factor TFIIH) or nuclease XPF‐ERCC1 are essential for transcription and other DNA repair pathways, respectively, offering a plausible explanation for the exacerbation and diversity of symptoms in these cases [185, 186, 187, 188, 189, 190]. However, mutations in the scaffolding protein XPA, exclusively associated with NER to date, also induce neurological abnormalities, suggesting that NER deficiency alone may suffice to trigger neurodegeneration [191, 192].

Strikingly, while the predisposition to cancer is characteristic of GG‐NER deficiency, TC‐NER‐deficient CS patients have no elevated risk of cancer but are defined by their premature aging features [7]. Mutations in TC‐NER genes *CSB* and *CSA* are causative for CS, a recessively inherited DNA repair disorder that presents a myriad of multiorgan system complications. The first hallmark features of CS arise mostly during early childhood, including developmental and neurological problems such as poor growth, microcephaly, cerebral calcifications, progressive neurodegeneration, neuronal defects due to demyelination, sensorineural deafness, premature aging, and a reduced life expectancy. Furthermore, CS patients often exhibit skin sensitivity to UV irradiation, liver and kidney dysfunction, visual impairments (corneal opacification and/or cataracts), and dental anomalies [118, 169, 193, 194, 195, 196, 197, 198, 199, 200, 201].

Globally, CS has a prevalence of 2–3 cases per million newborns and is categorized into three main types, based on symptom severity and age of onset [196, 198, 199, 202]. While Type I patients develop the first clinical signs in the first years after birth, the more severely affected Type II may develop clinical manifestations before birth, and Type III CS patients display late‐onset and milder clinical features [199, 201, 203]. A small subgroup of CS patients, bearing mutations in the CS proteins or the nucleases ERCC1‐XPF or XPG, may receive a diagnosis of COFS syndrome at birth. COFS represents the most severe form of CS and tragically leads to death within the first few years of life. In addition to the typical CS phenotype, COFS patients exhibit distinct facial features, developmental dysplasia of the hip, as well as cerebellar and mild renal hypoplasia [204, 205, 206]. The neurodegenerative disorder XP‐CS combines clinical characteristics of XP with those of CS [168]. In these cases, patients are born with deleterious mutations in TFIIH (*XPB* or *XPD* genes) or NER‐associated endonucleases (*XPF* or *XPG* genes) and display a range of symptoms, including short stature, progeria, progressive impairment of cognitive, hearing, and motor skills, and neurological problems, as well as extreme photosensitivity and skin pigmentation abnormalities. While CS‐like myelinopathy underlies the neurologic abnormalities, the cutaneous features in affected individuals resemble those observed in XP patients. The severity of symptoms can vary significantly dependent on the mutation in specific genes, as exemplified by mutations in ERCC1‐XPF, which can give rise to mild XP features up to the severe xeroderma pigmentosum F‐Ercc1 (XFE) progeroid syndrome. This XP‐CS‐like disorder is associated with more severe liver and kidney dysfunction and high blood pressure [178, 207, 208, 209]. At the other end of the clinical spectrum are UVSS patients who display only mild cutaneous symptoms [210, 211, 212]. Importantly, UVSS patients do not exhibit the severe progeroid signs or neurological abnormalities as in CS, despite an apparent similar cellular TC‐NER deficiency for bulky UV‐induced lesions. This peculiar phenotypic difference will be further discussed in section “DNA damage‐induced transcription stress is the main driver of CS”.

Another neurodegenerative pathology associated with NER mutations is trichothiodystrophy (TTD). In contrast to CS and XP, which are predominately caused by the loss of DNA repair function, TTD is thought to arise mainly from disrupted gene expression, resulting in dry, brittle, and sulfur‐deficient hair, short stature, intellectual disability, neurodevelopmental defects, ichthyotic skin, and premature aging. Among TTD patients, approximately half exhibit photosensitivity, which is caused by alterations in TFIIH helicases XPB and XPD, or in the smallest TFIIH subunit TTDA (GTF2H5), leading to defects in NER and transcription initiation [213, 214, 215, 216]. The other half of patients are classified as non‐photosensitive TTD (NPS‐TTD) patients and are not associated with NER deficiency. Strikingly, TTD features are most manifest in highly or termly differentiated tissue and are based on the instability of proteins involved in any step of gene transcription, ranging from mutations in transcription initiation factor TFIIE‐beta (*GTF2E2*) [217, 218] to splicing facilitating proteins such as RNF113A [219] and TTDN1 (or MPLKIP) [220, 221], or aminoacyl‐tRNA synthetases AARS1, MARS1, TARS1, and CARS1 [222, 223, 224].

## TC‐NER animal models

Unraveling the detrimental mechanisms underlying CS continues to be a challenge due to the limited number of patients, many of whom are compound heterozygotes, and the complexity and variability of the symptoms, thereby hindering indicative genotype–phenotype annotations. As the TC‐NER mechanism is evolutionarily conserved, simpler model systems, including mouse and *Caenorhabditis elegans* models, can help to investigate the consequences of unrepaired DNA lesions during development, aging, and tumorigenesis [22, 225]. Mouse models have been instrumental in drawing important connections between disease mutations and the physiological alterations that underlie pathological outcomes of the complete organism/multiple organs and tissues. These models often carry the same point mutations as identified in CS or TTD patients [226]. However, NER mouse disease models have also proven rather complex due to the fact that CS mouse models, except for *Xpg* ^*mut*^ and *Ercc1* ^*∆/−*^, do not fully replicate the severe features observed in human CS/COFS patients [22]. Instead, they generally exhibit mild features, including reduced body weight, UV sensitivity, and mild neurodegenerative changes. Why these mouse models lack a clear CS‐like phenotype remains elusive, although it was proposed that insufficient DNA lesions (and their consequent lesion‐stalled Pol II complexes, see below) were accumulating during their short lifespan. Indeed, by increasing the DNA damage load through targeting other DNA repair pathways as seen by backcrossing *Csa* ^*−/−*^ or *Csb* ^*m/m*^ mutant mice into *Xpa* ^*−*/−^ or *Xpc* ^*−/−*^ deficient mice, a severe aggravation of the mild CS phenotype was observed [197, 227, 228, 229, 230]. This concept is further supported by the recently generated TC‐NER‐deficient *Polr2a* ^K1268R/K1268R^ (ortholog of human *POLR2A* encoding RPB1) mice that also did not display a clear CS‐like phenotype [50]. However, *Polr2a* ^K1268R/K1268R^/*Xpa* ^−/−^ double mutant mice developed CS‐like characteristics including growth retardation and low body weight that worsened over time, leading to early death around 5–6 months. These exacerbated CS‐like phenotypes in mice suggest that DNA damage accumulation is a major source of the clinical features.

Importantly, the nematode *C. elegans* has proven to be an excellent model organism to study the cell‐specific consequences (see below) of NER‐ and TC‐NER deficiencies, highlighted by the ability to distinguish GG‐ or TC‐NER‐related genes through UV survival experiments at two different life stages of the worm [225, 231, 232, 233]. Moreover, in comparison to mice, *C. elegans* can more easily be genetically modified by CRISPR‐Cas9 or small interfering RNAs and has a very short regenerative cycle [234, 235]. This makes the nematode highly suitable for high‐throughput genetic screens to either identify synthetic lethal or suppressor genes, for rapid validation of newly identified factors and to assess their impact at the organism level. Moreover, its translucency allows direct *in vivo* live cell imaging, which previously revealed tissue‐specific NER activity [225]. Its versatility in (TC‐)NER research is illustrated by the development of a simple and fast assay to assess neuronal activity in response to TBLs [225] and by the peculiar observation that knocking out *xpa‐1* (ortholog of human *XPA*) partially alleviated neuronal defects caused by transcription stress [236]. This transcription stress is a consequence of prolonged TFIIH binding arising from *xpg‐1* and *xpf‐1* (orthologs of respectively *XPG* and *XPF*) deficiency. It is suggested that by removing XPA‐1, known to stabilize TFIIH at NER intermediates [140], prolonged TFIIH binding is suppressed and thereby partially mitigates transcription stress.

## DNA damage‐induced transcription stress is the main driver of CS

A pivotal question revolves around whether the symptoms of CS arise mainly from a TC‐NER deficiency or from a combination of pathogenic events driven by distinct mechanisms. Over time, the intricate nature of CS, XP, XP‐CS, and COFS has fueled numerous scientific debates and hypotheses regarding the diverse phenotypes associated with these conditions. Among these, a notable hypothesis challenges the conventional view of CS as a DNA repair disorder, suggesting it may be more aptly characterized as a transcription syndrome [237, 238, 239, 240]. This proposition stems from observations that repair‐deficient XP patients with mutations in genes like *XPA*, *XPF*, or *XPG*, who are also deficient in TC‐NER, lack the markedly more severe symptoms seen in CS patients. Therefore, it was proposed that symptoms in CS arise from transcriptional defects. This hypothesis is supported by the observation that loss of CSB function, both in patient‐derived CS cells and animal models, resulted in global gene expression changes in the absence of exogenous DNA damage, leading to the upregulation of inflammatory genes and the downregulation of neuronal genes [239, 241, 242]. This hypothesis, however, does not account for dysfunctional repair, and therefore the accumulation of stochastic endogenous DNA damage could also be the underlying cause of the transcriptional defects in CSB‐deficient cells.

The possible impact of endogenous DNA damage and its role in CS progression was corroborated in a recent study by Mulderrig *et al*. [243]. They generated a mouse model deficient in formaldehyde clearance enzyme ADH5 (*Adh5* ^*−/−*^) and CSB (*Csb* ^*m/m*^). This model presented features that faithfully replicate the phenotype of CS patients, including cachexia, neurodegeneration, and kidney failure. They provided evidence that by removing one of the aldehyde‐scavenging enzymes an excess of aldehyde‐induced ICLs was produced (Table 2), which could not be repaired in the absence of CSB. This observation shows that the canonical TC‐NER machinery is one of the repair pathways required to remove ICL‐derived TBLs and, importantly, that indeed unrepaired endogenously produced DNA damage, as a consequence of increased cellular aldehydes, is a major cause of CS. ICLs can be repaired by several pathways including the replication‐driven Fanconi anemia (FA) pathway (FA‐ICLR) [244] and replication‐independent repair mechanisms [245, 246, 247]. This pathway entails a bewilderingly complex genome surveillance mechanism, integrating different DNA repair processes and comprising a multitude of different repair proteins. Pathogenic mutations in any of the more than 20 FA‐ICLR genes give rise to the genetically and phenotypically heterogeneous cancer‐prone Fanconi anemia syndrome, further associated with congenital malformations and bone marrow failure. It thus appears that deficiencies in either of the two ICLR pathways gives rise to completely different phenotypes, with FA‐ICLR defects mainly affecting development and rapidly dividing tissue (bone marrow), whereas absence of TC‐ICLR predominantly affects post‐mitotic neurons. In section “Why are neurons more affected?”, we will further discuss tissue‐specific repair deficiencies. Recently it was shown that very rare cases of AMeD (aplastic anemia, mental retardation, and dwarfism) syndrome within the Japanese population was linked to combined pathogenic mutations in two aldehyde detoxifying genes, *ADH5* and, in this population, the common variant Rs671 of *ALDH2* [248]. Curiously, the complex phenotype of AMeD seems to be a combination of both FA‐ and CS‐specific features, triggering the suggestion that non‐detoxified aldehydes cause an excess of ICLs to levels that cannot be sufficiently cleared by FA‐ICLR and TC‐ICLR, further suggesting that endogenous DNA damage drives part of the CS features.

However, it should be noted that in addition to ICLs, aldehydes also cause other types of DNA lesions, including DPCs [249] (Table 2). Very recently, three back‐to‐back publications [34, 35, 36] showed that DPCs also cause strong transcriptional interference and that the major TC‐NER factors CSA and CSB play a crucial role in resolving DPC‐stalled Pol II. Strikingly, although both CSA and CSB were required to clear DPCs in a transcription‐dependent manner, the downstream NER factors do not play a role, suggesting that this entails a separate non‐canonical TC‐DPC repair pathway.

Another form of endogenous damage arises from oxidative DNA lesions (Table 2), induced by ROS formed as a byproduct of cellular respiration [250]. It has been reported that human and mouse cells carrying mutations in the *CSA* and *CSB* genes are hypersensitive to oxidative DNA damage [119, 242, 251, 252, 253, 254], suggesting that TC‐NER partially protects against oxidative DNA damage [255, 256]. However, *in vitro* studies showed that Pol II does not stall on the main non‐helix disturbing oxidative lesions [257], but does on a subset of more bulky oxidative products, such as cyclopurines [256]. The majority of non‐bulky oxidative lesions are removed by specific glycosylases within the BER pathway, leaving an abasic or apurinic/apyrimidinic (AP) site that is further processed into a SSB intermediate by downstream BER factors such as APE1, and subsequently repaired by PARP1, XRCC1, DNA polymerase β, and ligase III [31]. Notably, AP sites and SSBs formed as a consequence of glycosylase action stall transcription [258, 259] and recruit both CSB and downstream (TC‐) NER factors [254, 260, 261]. In these cases, TC‐NER responded to a BER‐mediated reaction intermediate rather than to the lesion itself.

In addition, Pol II transcription and CSB were found to promote the recruitment of downstream BER factors, pointing towards the existence of a TC‐BER pathway [31, 161, 254]. The significance of CSB in oxidative damage repair is underscored by a study using the induced pluripotent stem cells (iPCs) of CS patients, revealing defects in ROS response, elevated p53 levels, and cell death [262]. Moreover, *Csb* ^*m/m*^ mice showed progressive retinal degeneration, which severely exacerbated upon exposure to low doses of oxidative DNA damage [263]. Collectively, these findings strongly argue that the accumulation of endogenous TBLs in TC‐NER‐deficient CS cells is the main driver for the CS phenotype.

Peculiarly, although individuals with UVSS are TC‐NER‐deficient, they display only mild cutaneous UV sensitivity without progressive neurological decline [264]. These striking phenotypical differences may be partially attributed to the involvement of CS proteins in additional pathways such as transcription regulation, redox homeostasis, DSB and DPC repair, and possibly mitochondrial DNA repair [34, 35, 36, 265], for which a role for UVSSA has not been described yet. Supporting this hypothesis is the observation that CS cells, but not UVSS cells, are sensitive to oxidative DNA damage and DPCs [34, 119]. While one of the first UVSSA publications postulated that defective Pol II processing (i.e., ubiquitylation and degradation) might be the source of the strong CS phenotype [60, 266], another study reported actually the opposite, increased RPB1 degradation in CSB KO cells [51, 90], leaving the question of Pol II processing in CS cells controversial. However, recently it was shown that in CSA‐ or CSB‐deficient cells there is prolonged stalling of lesion‐bound Pol II as compared to TC‐NER‐proficient cells [162]. In contrast, in UVSSA‐deficient cells, lesion‐stalled Pol II is efficiently cleared from chromatin. Furthermore, additional data showed that CRL4^CSA^‐mediated ubiquitylation of Pol II (the loading of which requires the presence of CSB) and its subsequent chromatin extraction through VCP segregase are required for the clearance of stalled Pol II. This analysis revealed that UVSSA KO cells prevented the toxic effects of Pol II stalling through actively degrading the polymerase, since CSB and CRL4^CSA^ can still be recruited to ubiquitylate Pol II even in the absence of UVSSA. However, since UVSSA is required for downstream TC‐NER activity (e.g., TFIIH recruitment), TBLs remain, explaining UVSSA KO's hypersensitivity to TBL‐inducing agents and RRS defects. Together, these observations further fuel the hypothesis that lesion‐stalled Pol II rather than the lesion itself is the root cause for cellular toxicity in CS [7, 60].

Given the substantial overlap between the CS phenotype and the recognized hallmarks of aging, a hypothesis emerged that DNA damage‐induced transcription stress serves as an important molecular driver of aging [14]. Recent support for this idea comes from a study demonstrating that a larger fraction of elongating RNA Polymerases were stalled in liver cells obtained from 2‐year‐old mice as compared to isogenic young adult mice [267]. This stalling significantly compromised productive transcription, leading to a gene‐length‐dependent skewing of the transcriptional output. Importantly, a similar reduction in the expression of long genes was noted in young livers from repair‐deficient mice [268]. Since longer genes have a higher probability to acquire stochastic DNA damage, it is likely that Pol II stalls on endogenously generated lesions that gradually accumulate over time and are possibly difficult to repair. Indeed, endogenous DNA damage emerged as the root cause of the transcriptional stress observed in older animals, affecting pathways such as energy metabolism, autophagy, and immune function [267]. The stalling of Pol II during elongation might intuitively suggest a reduction in the elongation rate of Pol II. However, an independent study analyzing changes in genome‐wide transcription across various species during aging unveiled a surprising opposite trend: that transcription speed increases with age [269]. This effect correlated with a reduced accuracy in the transcription process, as evidenced by an increase in rare splicing events.

To elaborate on these two discoveries, a collaborative letter from both research teams proposed arguments on why both findings are complementary and ultimately lead to the observed cellular events, rather than being contradictory [270]. Firstly, Gyenis *et al*. [267] identified a general increase in transcriptional activity in aged liver cells, likely linked to a compensatory mechanism to counteract non‐productive transcription. This increase could shape a chromatin landscape that is more open and diffuse, which facilitates faster Pol II elongation. Secondly, it is possible that during the RNA‐seq procedure [269], transcripts of stalled Pol II are captured less efficiently than those in the elongation phase due to the retention of the RNA molecule by DNA–RNA hybrids. Both findings will have profound implications for genome maintenance in non‐replicating cells, which heavily rely on TC DNA repair [225]. Moreover, the reduction in nucleosome compaction is expected to increase the DNA damage burden as it exposes the DNA to damaging agents, including endogenous metabolic byproducts. The abundance of stalled Pol II will initiate TC repair mechanisms, which will be limited by the availability of repair‐associated proteins present in the cell. Collectively, these findings strongly support the notion that the buildup of unrepaired DNA damage is indeed the underlying cause of the transcription stress and cellular dysfunction, ultimately resulting in cell death observed during both normal aging and progeroid syndromes.

## Why are neurons more affected?

While all cell types respond to DNA damage, the extent to which they express DNA repair factors and initiate DNA repair varies between different cell types. Consequently, the implications of DNA damage on cell function and viability were found to be cell type‐, tissue‐, and organ‐specific [225, 233, 271]. Many progeroid syndromes caused by hereditary mutations in DNA repair genes manifest severe neuropathological symptoms [272, 273, 274, 275, 276, 277]. Single‐cell sequencing of human neurons indeed confirmed that neurons acquire somatic mutations during aging and that this accumulation is exacerbated in cells from individuals with neurodegenerative diseases [278]. Additionally, cancer survivors display structural and functional alterations in their brains along with premature aging as a consequence of genotoxic chemotherapy and radiotherapy treatments [14, 279, 280]. This prompted the question: why do post‐mitotic neurons demonstrate increased sensitivity to unrepaired DNA damage compared to other cell types?

We postulate that the observed sensitivity to DNA damage in neurons arises from interconnected multiple causes, ultimately leading to neuronal dysfunction and cell death. Firstly, post‐mitotic neurons lasting an organism's entire lifespan heavily depend on efficient DNA repair mechanisms to safeguard their genome integrity, as they don't benefit from the dilution of DNA damage through replication seen in actively dividing cells [14, 281]. Secondly, these post‐mitotic cells are devoid of several replication‐associated DNA repair processes, such as homologous recombination (HR) or FA‐ICLR, and thus have a limited repertoire to clear the genome. Interestingly, post‐mitotic neurons were found to circumvent these limitations by employing RNA‐templated HR [282, 283]. Moreover, a gene length Pearson correlation analysis revealed that the transcriptome of neurons is inclined towards longer transcripts when compared to non‐neuronal cell types [284]. These transcripts from relatively long genes are crucial for neuronal functioning and have a higher probability than shorter genes to acquire stochastic DNA damage from both genotoxic chemotherapeutics and cell‐intrinsic sources, such as spontaneous deamination, ROS and aldehyde metabolites. This, together with the absence of replication‐associated DNA damage clearance processes, makes neuronal cells particularly vulnerable to TBLs.

**Fig. 5:**
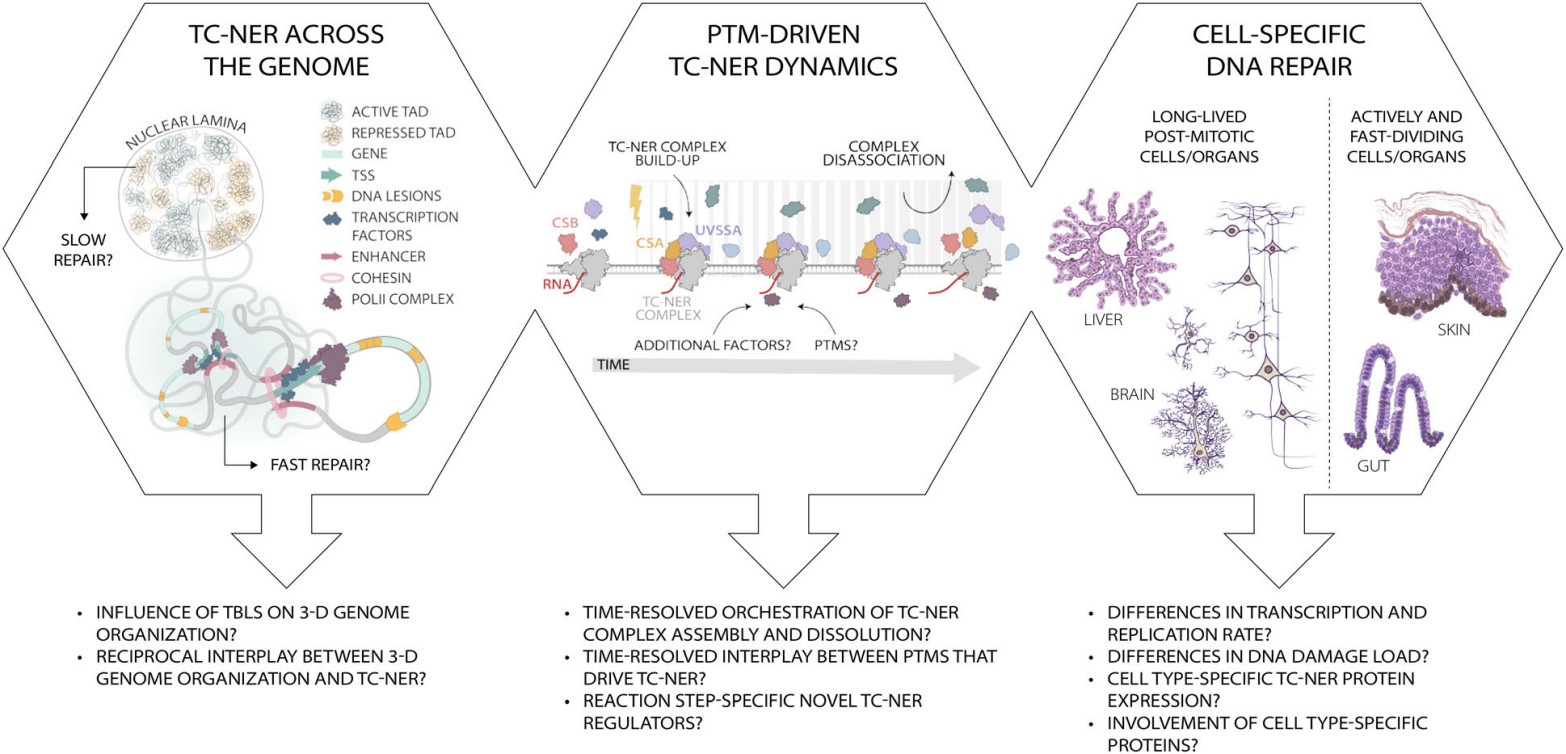
Future transcription‐coupled nucleotide excision repair (TC‐NER) research areas. From left to right: TC‐NER across the genome, depicting three‐dimensional (3D) chromatin organization of the nucleus clustered in topologically associating domains (TADs). Zoom‐in of an active TAD shows cohesion‐facilitated chromatin looping, bridging an enhancer region with the promoter region upstream of the transcription start site (TSS) of a gene. If and how the multi‐layered organization of chromatin facilitates and/or regulates TC‐NER is currently unknown. PTM‐driven TC‐NER dynamics is another unexplored topic, revolving around the question of TC‐NER complex buildup, disassociation, and reaction step‐specific protein interactors. Cell type‐specific DNA repair might exist between long‐lived post‐mitotic cells/organs such as liver and brain cells or actively and fast‐dividing cells/organs such as the skin or the gut.

Lastly, beyond DNA damage caused by environmental and endogenous genotoxic agents and transcription‐associated DNA breaks, neurons actively induce DNA breaks during neuronal activity to manipulate and regulate their (epi)genome, adding to the overall burden of DNA lesions in neurons [285, 286, 287, 288]. The role of these “programmed” DNA breaks in normal neuronal physiology and disease was recently explored by two concurrent studies conducted by Wu *et al*. [289] and Reid *et al*. [290]. Given the absence of genome replication in post‐mitotic neurons, both research groups identified locations of DNA synthesis associated with DNA repair by sequencing EdU‐incorporated DNA fragments, termed SAR‐seq (for: synthesis associated with repair sequencing) by Wu *et al*. [289] or Repair‐seq by Reid *et al*. [290]. In total, SAR‐seq revealed more than 55 000 peaks across the genome of iPC‐derived post‐mitotic glutamatergic neurons, which were highly reproducible and neuron‐specific. The comparison of SAR‐seq peaks to accessible chromatin regions measured by Assay for Transposase‐Accessible Chromatin using sequencing (ATAC‐seq) and to ChIP‐seq data with enhancer markers demonstrated that DNA‐repair‐associated synthesis is enriched in active enhancer regions. Notably, they discovered that 90% of super‐enhancers defining neuronal identity exhibited SAR‐seq peaks, in contrast to only 25% for conventional neuronal enhancers. Furthermore, Wu *et al*. [289] determined that SSBs were the primary source of DNA damage. Based on the enrichment of SSBs at C/G nucleotides and the high prevalence of CpG methylation of neuronal enhancers in post‐mitotic neurons compared with other cell types, the researchers revealed that SSBs were created as repair intermediate during active cytosine demethylation [288]. These findings align with results obtained by Reid *et al*. [290], who also identified a close association between DNA repair hotspots and sites of CpG methylation. Moreover, they found that evolutionary conserved elements of the human genome are enriched in DNA repair hotspots, suggesting that these regulatory regions may be disproportionately more protected. DNA repair, therefore, emerges not only as an emergency service for repairing stochastic breaks, but also as a fundamental process important for neuronal development, differentiation, and maintenance. Interestingly, they also found that DNA repair at neuronal enhancers is predominantly performed by long‐patch (LP‐) BER, a sub‐pathway that typically replaces 2–20 nucleotides, and that this pathway becomes more active upon suppression of short‐patch (SP‐) BER [289]. Although highly active in this situation, LP‐BER appears to be less efficient than SP‐BER; deficiencies in SP‐BER are linked to neurological diseases, indicating that LP‐BER cannot fully compensate for the loss of SP‐BER [276]. The prevalence of a specific DNA repair pathway in neurons is in line with the notion that the cell type, along with its developmental and differentiation state within an organism, influences the availability of specific DNA repair pathways [233, 291]. Recent work in *C. elegans* elaborated on whether these differentiation‐driven changes are a result of rewiring of the DDR, altered activity, or changes in expression levels and/or stability of specific DDR factors [225]. It was shown that the endonuclease XPF‐1 (worm ortholog of XPF)—important for NER, ICLR, and DSBR—displays a tissue‐specific response to UV damage, with differential contributions of either GG‐NER or TC‐NER activity in proliferating meiotic cells or post‐mitotic neurons respectively. Furthermore, tissue‐specific responses to DNA damage were shown to contribute to aging differences between organs [271]. In this study, rapid‐proliferating intestinal cells and post‐mitotic liver cells from progeroid repair‐deficient *Ercc1* ^*Δ/−*^ mouse mutants were systematically compared across tissue, stem cell, and organoid levels, revealing organ‐specific anti‐aging strategies. While intestinal cells favored apoptosis, liver cells relied on DNA repair pathways that specifically safeguard actively transcribed genes, ensuring sustained functionality and cell preservation. While initial research in highly differentiated cells such as neurons and hepatocytes argue for a cell type‐specific regulation of DNA repair, it is important to broaden the research question by investigating whether these findings also apply to the various cell types present in the brain, the gut, the liver, or the skin (Fig. 5).

## Future perspectives for TC‐NER research

Functional transcription regulating elements such as enhancers play a pivotal role in establishing chromatin loops and are integral to the three‐dimensional (3D) organization of chromosomes within the cell nucleus. For comprehensive insights into nuclear 3D genome organization, we recommend two outstanding reviews written by da Costa‐Nunes and Noordermeer [292] and Di Stefano and Cavalli [293]. In essence, individual chromosomes occupy distinct, evolutionary conserved chromosome territories within the nucleus. Between these territories, various chromosomal regions can congregate, forming either spatially active (A) or inactive (B) compartments, distinguished by unique GC‐content, chromatin marks, and gene density. At the sub‐megabase scale, the genome folds locally into topologically associating domains (TADs), characterized by enhanced intra‐domain chromatin contacts of multiple DNA loops. TAD formation is crucial for gene regulation, evident from the development of genetic diseases and cancer upon TAD disruption [294, 295]. In recent years, several studies pointed towards a fundamental role of 3D genome organization in DNA repair, particularly in actively transcribed regions [296, 297, 298, 299, 300]. Until now, most techniques capturing genome‐wide chromatin interactions (i.e., Hi‐C experiments) have been applied to study the 3D genome alterations upon DSB induction [297, 299, 300, 301]. Here, the formation of TAD‐sized structures was deemed essential for the compaction of DSB‐flanking chromatin during repair, thereby facilitating H2AX phosphorylation and DNA homology search. Moreover, TADs enclosing DDR genes with high R‐loop levels were found to cluster upon local DSB induction, suggesting that R‐loops may promote compartmentalization and regulation of DDR [300]. The most recent discovery includes the genome‐wide mapping of AFB1‐dG repair across multiple genome organization levels [302]. Here, AFB1 adducts were shown to be predominantly repaired by TC‐NER in genes (a) close to the nuclear center, (b) part of A compartment, (c) at the TAD boundaries, and (d) at the anchor regions of chromatin loops.

Interestingly, recent evidence suggests that the transcriptional process and/or its constituent proteins, along with the response to DNA damage, influence the 3D genome structure as well, indicating a reciprocal interaction between fine‐scale genome organization, transcription, and DDR [303, 304, 305]. Moreover, *in silico* predictions have revealed that highly interacting genomic regions, such as enhancers and super‐enhancers, are typically transcriptionally active regions and tend to be more fragile than other parts of the genome [306]. In addition, most identified HIRs have shown increased binding of DNA repair factors, suggesting a significant role for DNA repair in 3D genome organization and transcription, thereby preventing detrimental genome rearrangements. With spatial chromosome folding facilitating transcription, it is intriguing to speculate on how the multi‐layered organization of the nucleus will change upon Pol II encountering a TBL and whether chromatin folding will contribute to TC‐NER initiation, UV‐induced downregulation of transcription [81, 124] or downstream processes such as lesion removal and transcription restart (Fig. 5). One of the main difficulties in investigating UV‐induced genome rearrangements is the inability to localize the site of damage due to the stochastic nature of UV lesions. While DSB researchers circumvented this problem by generating the human DIvA (DSB inducible via AsiSI) cell line, which induces about a hundred euchromatic DSBs located at the AsiSI restriction sites [307], such a tool does not exist for bulky TC‐NER‐inducing lesions yet. A direction one might consider is utilizing the sequence‐specific targeting of the CRISPR‐Cas9 system. Bioengineering of Cas9 fusion proteins has provided a range of possibilities, including targeting transcriptional effectors or epigenetic modifiers to specific loci in the genome [308]. Coupling an alkylating enzyme, which modifies the DNA structure [160], or an AP‐endonuclease to create AP sites to a (catalytic dead) Cas9 protein coupled to multi‐target guide RNAs [309] might provide a tool to create TBLs at specific loci. Here, lesion recognition would be induced by releasing cells from reversible transcription inhibitors, allowing Pol II to synchronously enter the gene body [310] and providing the time control needed for Hi‐C experiments, monitoring binding of specific factors, or identifying local chromatin changes.

Beyond structured chromatin organization, nuclear subdomains can be shaped by physicochemical forces, leading to the demixing of liquids and the formation of membraneless organelles and protein condensates. Through phase separation (PS), cells transiently confine specific functional elements to create structures like the nucleolus or paraspeckles, or modulate various cellular processes including gene expression and cell signaling [311, 312, 313]. Dysregulation of PS has been associated with the accumulation of protein aggregates that transition into solid‐like structures in pathologies such as amyotrophic lateral sclerosis, frontotemporal dementia, and cancer [311, 314, 315]. Similar to genome organization, PS has emerged as a new area of interest in the DDR field, with numerous studies providing compelling evidence for its involvement in transcription and DNA repair [316, 317, 318, 319, 320, 321, 322, 323, 324, 325]. Summarizing all findings is beyond the scope of this review; we recommend consulting specific reviews on this topic authored by e.g., [326, 327, 328]. Proteins and nucleic acids involved in PS often contain intrinsically disordered regions (IDRs) or low‐complexity domains [326, 329]. Additionally, PS is susceptible to environmental changes and is influenced by PTMs including methylation, phosphorylation, ubiquitylation, SUMOylation, and PARylation [330, 331, 332]. Although PS has not been described for TC‐NER yet, this physicochemical process contributes to the transition from the initiation phase to the elongation phase of Pol II [318, 333], and may also promote the recruitment, stabilization, and spatiotemporal control of TC‐NER complexes. Possible candidates partaking in PS during TC‐NER could be nascent RNA or the N‐terminal unstructured part of CSB or the mid‐region of UVSSA (both contain IDRs). With many aspects of PS still unknown, studies performed in a controlled *in vitro* transcription system will be essential for understanding the basics of this process before transitioning to a cellular context and deciphering its role during the TC‐NER process.

## Conclusion

In this review, we elaborated not only on the most recent pioneering work on TC‐NER, but also highlighted that DNA repair in transcriptionally active genes is essential for maintaining cellular function and protecting cells from various types of lesions, including endogenous damage. Over the years, our understanding of the initial CSB‐CSA‐UVSSA mechanism evolved into a more complex process, requiring additional protein factors and the dynamic regulation by PTM's. Moreover, the scope of TC‐NER's function grew with the realization that restoring the DNA template is as important as preventing TBL‐caused transcription stress [7]. With this knowledge we now look further, questioning whether an overarching or canonical TC‐NER model exists, or whether variations of this process take place depending on the location of a TBL within a gene body, the expression rate of a specific gene, genomic location, chromatin environment, or the type of cell. While each technology discussed here has contributed greatly to solving the TC‐NER puzzle, acknowledging their limitations and integrating the different techniques will be the key to obtaining further mechanistic insights. Furthermore, future experimental setups should consider adding the component of time, as the TC‐NER complex might not be a static complex and regulates its repair efficiency by recruiting other proteins at various time points post‐UV (Fig. 5).

## Conflict of interest

The authors declare no conflict of interest.

## Author contributions

DALS, AP, and WV wrote the review, and YJP provided the artwork.
